# Determinative Developmental Cell Lineages Are Robust to Cell Deaths

**DOI:** 10.1371/journal.pgen.1004501

**Published:** 2014-07-24

**Authors:** Jian-Rong Yang, Shuxiang Ruan, Jianzhi Zhang

**Affiliations:** Department of Ecology and Evolutionary Biology, University of Michigan, Ann Arbor, Michigan, United States of America; Yale University, United States of America

## Abstract

All forms of life are confronted with environmental and genetic perturbations, making phenotypic robustness an important characteristic of life. Although development has long been viewed as a key component of phenotypic robustness, the underlying mechanism is unclear. Here we report that the determinative developmental cell lineages of two protostomes and one deuterostome are structured such that the resulting cellular compositions of the organisms are only modestly affected by cell deaths. Several features of the cell lineages, including their shallowness, topology, early ontogenic appearances of rare cells, and non-clonality of most cell types, underlie the robustness. Simple simulations of cell lineage evolution demonstrate the possibility that the observed robustness arose as an adaptation in the face of random cell deaths in development. These results reveal general organizing principles of determinative developmental cell lineages and a conceptually new mechanism of phenotypic robustness, both of which have important implications for development and evolution.

## Author Summary

It is widely believed that development plays an important role in the phenotypic robustness of organisms to environmental and genetic perturbations. But, the developmental process and cell fate are largely predetermined and fixed in some species, including for example mollusks, annelids, tunicates, and nematodes. How these organisms deal with perturbations that cause cell deaths in ontogenesis has been a long-standing puzzle. We propose and demonstrate that the developmental cell lineages of these species are structured such that the resulting cellular compositions of the organisms are only moderately affected by cell deaths. A series of highly nonrandom features of the cell lineages underlie their developmental robustness and these features likely originated as adaptations in the face of various disturbances during development. Our findings reveal important organizing principles of determinative developmental cell lineages and a conceptually new mechanism of phenotypic robustness, which have broad implications for development and evolution.

## Introduction

Phenotypic robustness, often referred to as canalization, is the phenomenon that a phenotypic trait is invariant in the face of environmental or genetic perturbations [1]–[7]. Phenotypic robustness allows the maintenance of high fitness even under suboptimal conditions, which are not uncommon in nature [1]–[7]. Phenotypic robustness may also facilitate adaptation under certain conditions [8]. The genetic basis of phenotypic robustness has been of long-standing interest, and several underlying mechanisms have been elucidated [2], [3], [5]. For instance, capacitors such as molecular chaperones can buffer the disturbances from stressful environments and deleterious mutations; phenotypic variance is exposed upon the removal of capacitors [9], [10]. Functional redundancy in genetic systems is another cause of robustness because it renders the phenotype of an organism relatively invariant to the loss of a genetic component. Such redundancies are known to exist at both the individual gene level (e.g., between duplicate genes) [11] and the systems level (e.g., between alternative metabolic pathways) [5], [12]. Several evolutionary mechanisms explain the origin and maintenance of such functional redundancies and the resulting robustness [12]–[15]. Other proposed mechanisms of robustness include expression regulation via transcriptional regulatory networks [16], posttranscriptional regulation by microRNA [17], [18], and certain feedback/feed-forward circuits in signaling among cells [19].

It has long been recognized that ontogenesis, or the development of an organism from a fertilized egg to an adult, is a key component of phenotypic robustness [1]. But the mechanism underlying the ontogenic robustness is not well understood. Regulative development, where rescuing processes may be triggered in response to cell deaths caused by environmental or genetic perturbations, could ensure ontogenic robustness. However, regulative development usually accompanies massive cell rearrangements and migration before or during cell fate specification [20], which is not a desirable feature in species or tissues that have short developmental time, let alone the complex genetic or cell-cell communication network required for the regulation. In fact, no embryo displays only regulative development [20]. Even in largely regulative embryos, one finds determinative (also known as mosaic) development [20], where the developmental process and cell fate are fixed. In invertebrate embryos, especially those of mollusks [21], annelids [22], tunicates [23], and nematodes [24], [25], determinative development is extensively observed [20]. How do these species deal with environmental or genetic perturbations in ontogenesis? To answer this question, we investigate the ontogenic robustness of three invertebrates dominated by determinative development, using developmental cell lineages that describe the exact genealogical relations of all cells of an individual embryo or adult. We show that the determinative development of these invertebrates is highly robust to two types of cell death, which approximate the effects of random environmental disturbances (or somatic mutations) and genetic disturbances (i.e., germline mutations), respectively. We identify multiple extremely nonrandom features of the cell lineages that explain the ontogenic robustness, and show by evolutionary simulation that this characteristic can arise as an adaptation to certain disturbances in ontogenesis.

## Results

### Quantifying the robustness of determinative developmental cell lineages

**Figure 1:**
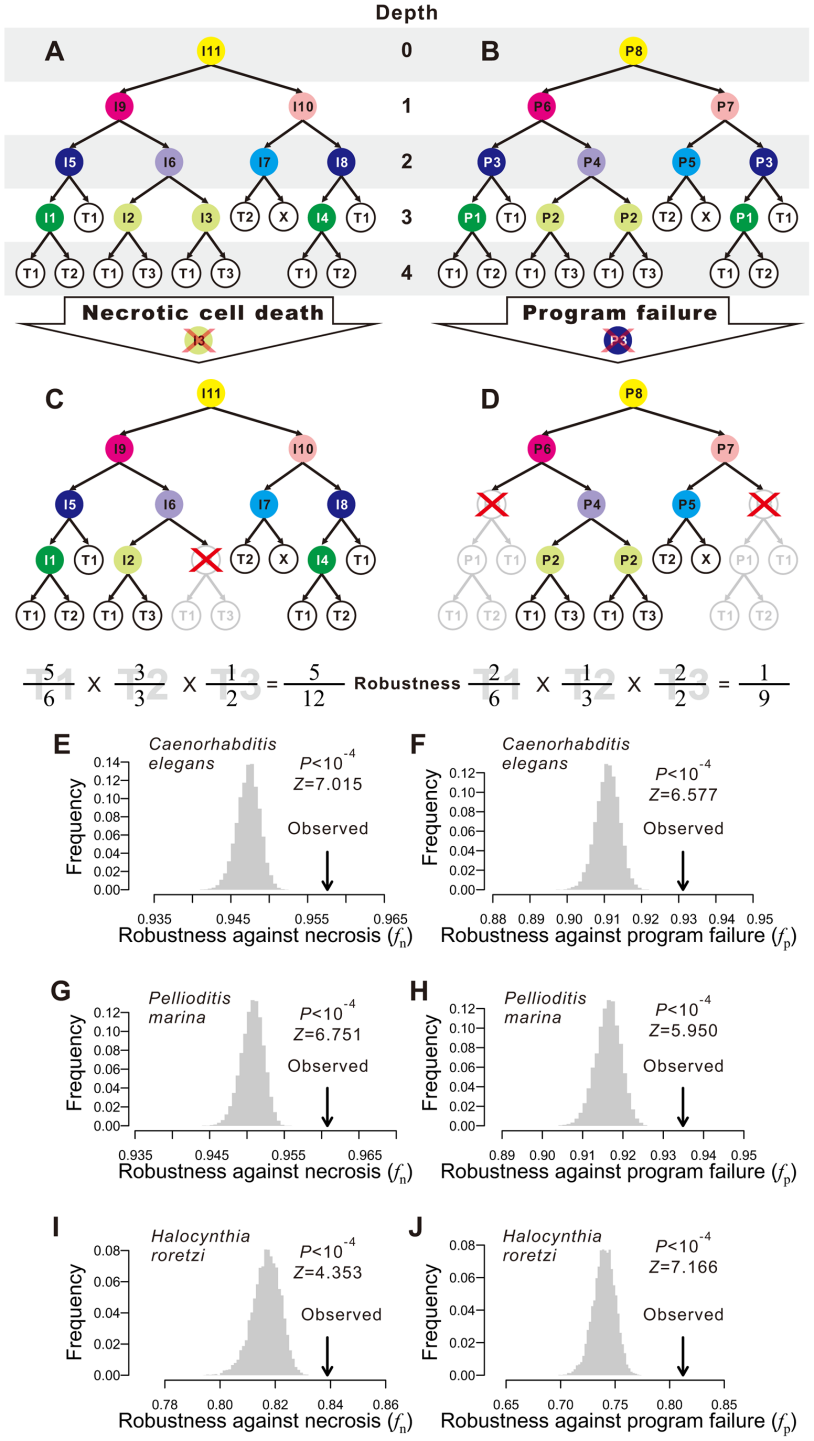
Animal developmental cell lineages are robust to necrosis and program failure. (A) A hypothetical cell lineage. Internal cells are prefixed with “I” and terminal cells are prefixed with “T”. Terminal cells belonging to the same cell type have the same name. Internal cells are colored according to their cell division programs. (B) The same cell lineage showing division programs for internal cells. Internal cells having the same division programs share the same color and program name (prefixed by “P”). (C) An example showing robustness calculation upon a necrotic cell depth. The internal cell I3 dies, which causes the loss of I3 as well as all of its direct and indirect descendant cells. Robustness is calculated by the product of the fraction of live terminal cells of each cell type. (D) An example showing robustness calculation upon a program failure. The failure of program P3 results in the loss of all descendant cells of internal cells that use P3. Robustness is calculated by the product of the fraction of live terminal cells of each cell type. (E–F) The *Caenorhabditis elegans* developmental cell lineage is more robust than the corresponding random lineages in the presence of (E) necrosis or (F) program failure. The grey bars show the frequency distribution of the robustness of 10,000 random lineages, whereas the arrow indicates the robustness of the *C. elegans* cell lineage. The random lineages are generated by randomly coalescing the terminal cells of the *C. elegans* lineage. *P*-value indicates the probability that a randomly generated lineage is more robust than the real lineage. *Z*-score is the number of standard deviations (of the random lineages) by which the observation deviates from the mean of the random lineages. (G–H) The *Pellioditis marina* cell lineage is more robust than the corresponding random lineages in the presence of (G) necrosis or (H) program failure. (I–J) The *Halocynthia roretzi* cell lineage is more robust than the corresponding random lineages in the presence of (I) necrosis or (J) program failure.

A typical developmental cell lineage takes the form of a binary tree composed of nodes and branches (**Fig. 1A**). The nodes represent cells, whereas the branches show descendant relationships among cells. There are two categories of nodes: terminal and internal. Terminal nodes represent cells at the final stage of the developmental process represented by the cell lineage, which may or may not be the final stage of development. Terminal cells are the ultimate product of the ontogenesis represented by the cell lineage. By contrast, internal nodes represent direct or indirect progenitors of the terminal cells, which are produced through differentiation and proliferation of the internal nodes. Here we consider only those cell lineages that start from the zygote.

In organisms such as the nematode worm *Caenorhabditis elegans*, cell fate determination is generally autonomous such that dead or degenerated cells are rarely replaced or compensated by other cells [25]. In other words, the cell lineage and the identity of each cell in the lineage are essentially invariable among individuals. We first classify the terminal cells in a cell lineage into different functional types [25], [26]; cells of different types perform distinct physiological functions whereas cells of the same type perform similar functions. From a cellular perspective, the ultimate consequences of both environmental and genetic disturbances to ontogenesis may be largely represented by the loss of certain terminal cells, although other consequences also exist (see Discussion). Because of the distinct functions of different terminal cell types, it is reasonable to assume, to a first approximation, that the probability of an organism to survive and reproduce is determined by the product of the weighted fraction of live terminal cells of each cell type. That is,
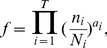
(1)where *T* is the total number of terminal cell types except apoptotic cells, *N_i_* is the number of terminal cells of type *i* in the absence of any disturbance, *n_i_* (*n_i_*≤*N_i_*) is the actual number of live terminal cells of type *i*, and *a_i_*≥1 is an exponent reflecting the relative importance of cell type *i* to organismal growth and reproduction. For simplicity, we describe our results using *a_i_* = 1 for all *i*. Using other *a_i_* values yields similar results (**Figs. S1A–F**). Here *f* can be viewed as a measure of developmental robustness to cell deaths. While *f* is likely a component of Darwinian fitness, it is not equivalent to fitness.

Impacts of environmental and genetic perturbations on cell lineages manifest as the loss of internal and terminal cells. When an internal cell dies, all of its direct and indirect descendant cells are regarded as lost. We consider two types of perturbation. The first type is referred to as necrosis [27] or simply random cell death. This type of lineal perturbation mimics environmental disturbances or somatic mutations that lead to accidental deaths of individual cells. Note that our use of necrosis is different from that in some literature where it also includes cell death caused by germline mutations [27]. The second type of perturbation is referred to as division program failure (see **Fig. 1B** and below for the definition of cell division programs), which mimics germline mutations that cause the deaths of all cells that use a particular genetic program for cell division. We consider all internal cells that use the failed program to be arrested, resulting in the loss of all direct and indirect descendants of these internal cells. The two types of perturbation only approximate environmental disturbances (and somatic mutations) and genetic disturbances (i.e., germline mutations), respectively, because of several kinds of exceptions (see Discussion). Let *f*_n_ and *f*_p_ be the *f* value in the presence of necrosis and program failure, respectively. To estimate *f*_n_, we first calculate *f* when one non-root cell and all of its descendants are removed (**Fig. 1C**). We repeat this process for every non-root cell in the lineage and calculate the mean resulting *f*, which is the expected lineage robustness to the death of a randomly chosen non-root cell. A real cell lineage is said to be robust to necrosis if its *f*_n_ is significantly higher than that expected from a randomized cell lineage that produces the same terminal cells.

To estimate *f*_p_, we follow a previous definition of cell division programs [26]. Every cell division in the lineage produces two daughter cells from a parental cell. The program used in the division is defined entirely by the types of the daughter cells. Here, a daughter cell may be terminal or internal, meaning that its type may be a terminal cell type or a division program (see node colors in **Figs. 1A, B**). Thus, two internal cells that give rise to the same types of daughter cells use the same program. For example, node I5 and I8 in **Fig. 1A** both use the program P3 (**Fig. 1B**). This definition is supported by the observation that the transcriptome of a cell is largely determined by the cell fate rather than the lineal history [28]. We traverse the entire cell lineage to define all division programs. If one program fails, all internal cells that use the program and all of their descendant cells are lost (**Fig. 1D**). We thus estimate *f*_p_ by calculating the expected *f* using Eq. (1), with a specific per-program rate of failure being 1 over the number of internal cells (see Materials and Methods). A cell lineage is said to be robust to program failure if its *f*_p_ is significantly greater than that expected from a randomized cell lineage that produces the same terminal cells.

### Determinative developmental cell lineages are robust to both necrosis and program failure

We used determinative developmental cell lineages starting from the zygote and up to a >100-cell stage. To our knowledge, such lineages have been completely described in only three animal species: *Caenorhabditis elegans*, *Pellioditis marina*, and *Halocynthia roretzi*. Previously reported developmental cell lineages of other species are incomplete in internal or terminal cells on the lineage tree, their mother/daughter relationship, and/or functional categorization of terminal cells, and thus cannot be analyzed here (**Table S2**). Among the three species to be analyzed here, the nematode *C. elegans* is the first animal with its developmental cell lineage mapped at the single cell resolution [25]. Here we use the *C. elegans* cell lineage producing 671 terminal cells during the hermaphrodite embryogenesis, and categorize the terminal cells by standard anatomical descriptions [26], [29]. *P. marina* is another nematode, but lives in the sea. The cell lineage of *P. marina*, followed up to muscle contraction, has 638 terminal cells [24], [26]. *H. roretzi*, commonly known as the sea squirt, is an ascidian. We use the cell lineage of *H. roretzi* up to the 110-cell stage in this study [26], [30]. Hence, our analysis includes representatives from both Protostomia (*C. elegans* and *P. marina*) and Deuterostomia (*H. roretzi*), the two subgroups of triploblastic animals.

We estimated *f*_n_ and *f*_p_ of each of the three cell lineages by considering necrosis (**Figs. 1E, G, I**) and program failure (**Figs. 1F, H, J**), respectively. To compare with each real lineage, we generated 10,000 random cell lineages under the assumption that, at any developmental stage, all terminal cells have the same probability of cell division. Computationally, each of these random lineages was created by randomly coalescing the terminal cells of the real lineage exactly two cells at a time (**Fig. S2**). We found that *f*_n_ and *f*_p_ are significantly greater in each of the three real lineages than in their corresponding random lineages (*P*<0.0001; **Figs. 1E**–**J**), demonstrating that the three animal cell lineages are robust to both necrosis and program failure. The reduction in *f* (from 1) caused by necrosis is on average 19.6, 20.4, and 12.1% smaller in the three real lineages, compared with their respective random lineages. The corresponding numbers are 22.8, 22.2, and 27.8% for program failure. If the terminal cells of the same functional type are not equally important [31], one could divide a cell type into subtypes when estimating *f*_n_ and *f*_p_, which would also result in more cell division programs. To evaluate the impact of considering subtypes of terminal cells and division programs on our results, we divided *C. elegans* neurons into five subtypes [26] and altered the definition of division programs correspondingly. The results, however, are qualitatively unaltered (**Figs. S1G, H**). We also examined an expanded hermaphroditic *C. elegans* cell lineage that includes post-embryonic cells, totaling 937 terminal cells, and the results are similar (**Figs. S1I, J**). One assumption made in our analysis is that all cells (or programs) have the same probability of necrosis (or failure). To investigate the impact of this assumption, we allowed different cells (or programs) to have different rates of necrosis (or failure) that follow an exponential distribution with the mean of the distribution identical to the constant rate used above. The obtained results are, however, similar (**Fig. S1K, L**), suggesting that our results are not sensitive to the assumption of equal necrosis (or program failure) rates. Because the overall results from the three species are similar, from now on, we present our findings from *C. elegans* in the main figures and those from the other two species in the accompanying supplementary figures.

### Shallowness of the cell lineages contributes to their robustness

To understand the underlying mechanisms of the observed lineage robustness to necrosis and program failure, we examined various characteristics of both the real and random cell lineages. Let us define the depth of a cell in a cell lineage by the number of cell divisions required to generate the cell from the root, which is the zygote in the lineages analyzed here. To a terminal cell, the smaller its depth, the lower the probability of its loss, because each cell division carries risks of necrosis and program failure. Hence, reducing the depths of terminal cells should improve *f*.

**Figure 2:**
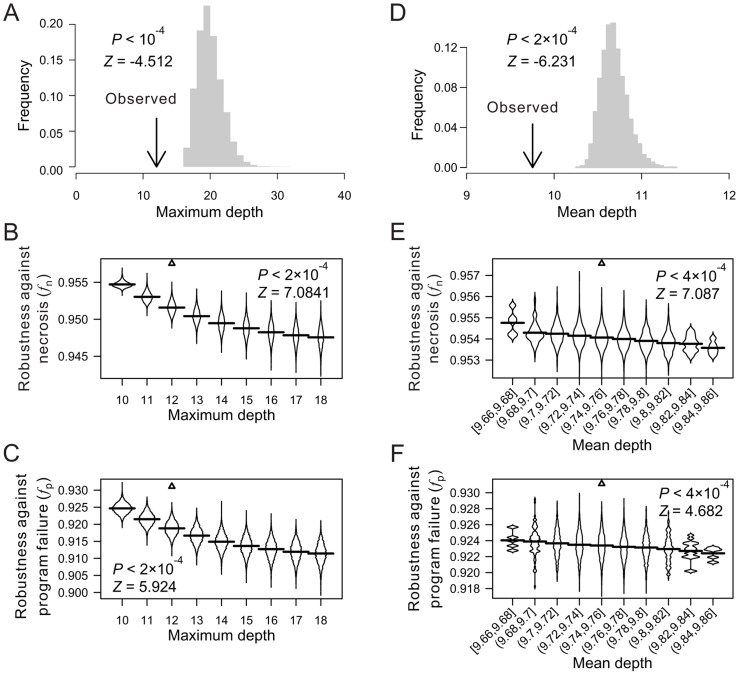
Low depths of terminal cells improve the robustness of the *C. elegans* lineage to necrosis and program failure. (A) Frequency distribution of the maximum cell depth in 10,000 lineages (grey bars), which are generated by random coalescence of the terminal cells of the *C. elegans* lineage. The arrow indicates the observed maximum cell depth in the *C. elegans* lineage. *P*-value is the probability that the maximum depth of a random lineage is smaller than that of *C. elegans*. *Z*-score is the number of standard deviations by which the observation deviates from the mean of the random lineages. (B–C) Violin plot for the robustness of randomly generated lineages with defined maximum depths in the presence of (B) necrosis or (C) program failure. Each violin is essentially a horizontal histogram showing the relative probability densities of different robustness of random lineages with the indicated maximum depth. The horizontal line in each violin plot shows the mean value. The real lineage is shown by a triangle. *P*-value is the probability that the robustness of a random lineage (with the same maximum depth as that of *C. elegans*) is higher than that of *C. elegans*. *Z*-score is the number of standard deviations by which the observation deviates from the mean of the random lineages. (D) Frequency distribution of the mean terminal cell depth in 5,000 lineages (grey bars), which are generated by random coalescence of the terminal cells of the *C. elegans* lineage with the requirement that the maximum depth is the same as in *C. elegans*. The arrow indicates the observed mean depth in the *C. elegans* lineage. *P*-value is the probability that the mean depth is smaller in a random lineage than in *C. elegans* when their maximum depths are the same. (E–F) Violin plot for the robustness of randomly generated lineages with the maximum depth equal to that of *C. elegans* and defined mean depths, in the presence of (E) necrosis or (F) program failure. The real lineage is indicated by a triangle. *P*-value is the probability that the robustness is higher in a random lineage (with the same maximum depth and similar mean depth as those of *C. elegans*) than in *C. elegans*. *Z*-score is the number of standard deviations by which the observation deviates from the mean of the random lineages.

Let us define the maximum depth of a cell lineage by the largest depth among all terminal cells in the lineage. Indeed, the maximum depth is significantly smaller in the three real lineages, compared with their corresponding random lineages (**Fig. 2A**; **Figs. S3A, B**). The theoretical minimum of the maximum depth of a cell lineage with a total of *L* terminal cells is [log_2_ *L*], where the square bracket represents the minimal integer that is no smaller than the number inside. The theoretical minimum of the maximum depth is 10, 10, and 7 for the three real lineages, respectively. The observed maximum depth is 12, 11, and 7, respectively, indicating that the real maximum depth is either identical to or close to the theoretical minimum. By calculating *f*_n_ and *f*_p_ of random lineages with different maximum depths, we found that, on average, *f*_n_ and *f*_p_ both increase as the maximum depth decreases (**Figs. 2B, C**; **Figs. S3C–F**), suggesting that reducing the maximum depth of a cell lineage tends to increase its robustness to necrosis and program failure. Intriguingly, the real lineages have significantly greater *f*_n_ and *f*_p_ than the random lineages with the same maximum depths (**Figs. 2B, C**; **Figs. S3C–F**), revealing the presence of additional factors that contribute to the high *f*_n_ and *f*_p_ of the real lineages.

We found the mean depth of all terminal cells in each of the three real lineages to be significantly smaller than that of their corresponding random lineages even when the maximum depths of these random lineages are fixed at the observed values (**Fig. 2D**; **Figs. S3G, H**). For each real lineage, we then generated 10,000 random lineages that have the maximum depth identical to the observed value and the mean depth similar to the observed value (See Materials and Methods). The mean depth is found to impact *f*_n_ and *f*_p_ negatively even when the maximum depth is fixed (**Figs. 2E, F**; **Figs. S3I**–**L**), confirming that a smaller-than-expected mean depth given the maximum depth is another contributor to the high *f*_n_ and *f*_p_ of the real lineages. Nonetheless, the real lineages still have greater *f*_n_ and *f*_p_ than the random lineages of the same maximum and mean depths (**Figs. 2E, F** **; Figs. S3I–L**), suggesting that additional factors contribute to the high *f*_n_ and *f*_p_ of the real lineages.

### Lineal topology and organization of terminal cells contribute to robustness

**Figure 3:**
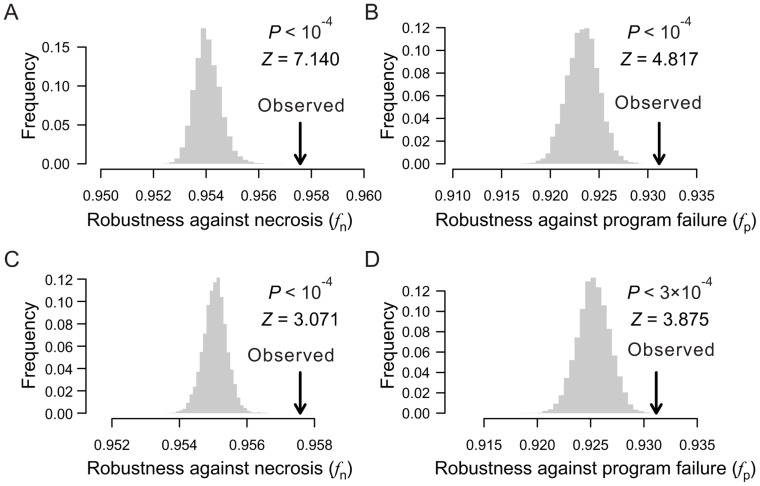
Lineal topology and terminal cell organization contribute to the robustness of the *C. elegans* lineage. (A–B) Frequency distribution of the robustness of 10,000 random lineages (grey bars) in the presence of (A) necrosis or (B) program failure. These random lineages have exactly the same depths as in *C. elegans* for all terminal cells but have randomized topologies. The arrow indicates the robustness of the *C. elegans* lineage. *P*-value is the probability that a random lineage above created is more robust than the *C. elegans* lineage. *Z*-score is the number of standard deviations by which the observation deviates from the mean of the random lineages. (C–D) Frequency distribution of the robustness of 10,000 random lineages (grey bars) in the presence of (A) necrosis or (B) program failure. These random lineages have exactly the same topology as the *C. elegans* lineage but have their terminal cells randomly relabeled. The arrow indicates the robustness of the *C. elegans* lineage. *P*-value is the probability that a random lineage above created is more robust than the *C. elegans* lineage. *Z*-score is the number of standard deviations by which the observation deviates from the mean of the random lineages.

When the depths of all terminal cells are fixed, the only thing in a cell lineage that can vary is the lineal topology. Here, the term “topology” is equivalent to that in phylogenetics, including both the lineage tree structure when the terminal cells are unlabeled and the arrangement of the labeled terminal cells given the tree structure. To test the impact of lineal topology on robustness, for each real lineage, we generated 10,000 random lineages with varying topologies but the same depth distribution as in the real lineage for all terminal cells. Again, *f*_n_ and *f*_p_ are significantly greater in the real lineages than in their respective randomized lineages (**Figs. 3A, B**; **Figs. S4A–D**), demonstrating the contribution of lineal topology to the high robustness of real lineages.

Furthermore, *f*_n_ and *f*_p_ are significantly greater in each real lineage than in its corresponding random lineages that share the same tree structure, which were generated by relabelling the terminal cells in the real lineage (**Figs. 3C, D**; **Figs. S4E–H**). Thus, the organization of terminal cells contributes to the robustness of the real lineages.

### Early appearances of rare cells improve robustness

**Figure 4:**
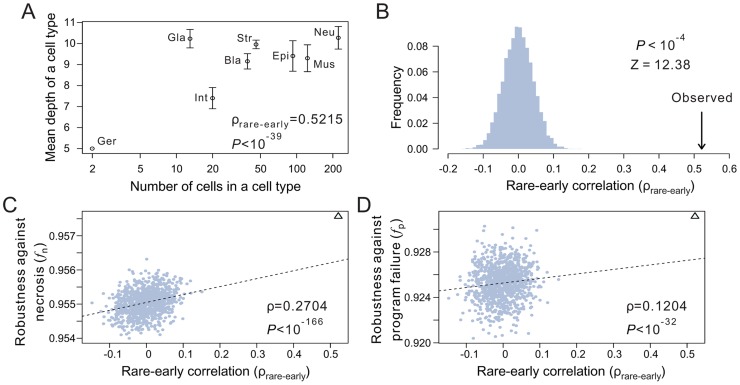
The tendency for rare cells to have low depths improves the robustness of the *C. elegans* lineage. (A) Positive correlation between the depth of a terminal cell and its cell type size. Spearman's rank correlation (ρ) for the original unbinned data and the associated *P*-value are presented. Error bars show one standard deviation of the depth within a cell type. Bla, blast; Epi, epithelial; Ger, germ; Gla, gland; Int, intestinal; Mus, muscle; Str, neural structural; Neu, neuron. The rare-early correlation remains strong even when the germ cells are removed (ρ = 0.515, *P*<10^−38^). (B) Frequency distribution of the rare-early correlation coefficient from 10,000 random lineages that have the same topology as that of *C. elegans* but have their terminal cells randomly relabeled. The arrow indicates the correlation coefficient for the *C. elegans* lineage. *P*-value is the probability that a random lineage above generated has a higher rare-early correlation than that observed in *C. elegans*. *Z*-score is the number of standard deviations by which the observed correlation deviates from the expected correlation of the random lineages with the same topology. (C–D) The stronger the rare-early correlation (ρ_rare-early_) in a random lineage, the higher the robustness of the lineage in the presence of (C) necrosis or (D) program failure. Although 10,000 random lineages are generated, for clarity, only 1000 are shown (grey dots). The dashed line is the linear least-square regression of these 1000 dots. The rank correlation between ρ_rare-early_ and robustness, as well as the associated *P*-value, are calculated from all 10,000 lineages. The *C. elegans* lineage is represented by a triangle.

What features of the terminal cell organization underlie the high robustness of the real lineages? As mentioned, terminal cells have been classified into functional types. We define the size of a cell type by the number of terminal cells belonging to the type. Cells of large cell types are referred to as common cells, whereas those of small cell types are referred to as rare cells. According to Eq. (1), loss of a rare cell has a larger adverse impact on *f* than the loss of a common cell. Because a low-depth terminal cell is less likely than a high-depth terminal cell to be lost, one strategy to improve *f*, given the lineage tree structure, is to arrange the terminal cells in such a way that the rare cells have relatively low depths and common cells have relatively high depths. Indeed, in each of the three real lineages, a positive correlation exists between the depth of a terminal cell and the size of its cell type (see binned results in **Fig. 4A**; **Figs. S5A, B**). This correlation (ρ_rare-early_) is significantly stronger than the chance expectation, which is calculated using 10,000 lineages constructed by randomly relabelling the terminal cells of each real lineage (**Fig. 4B**; **Figs. S5C, D**). A comparison among the random lineages shows that *f*_n_ and, to a much lesser degree, *f*_p_ increase with ρ_rare-early_ (**Figs. 4C, D**; **Figs. S5E–H**), confirming the prediction that early appearances of rare cells in a lineage render the lineage more robust.

The above analysis depends critically on the classification of terminal cells. For instance, if the late-appearing neuron cells are divided into many subtypes, the rare-early correlation would be weakened. It is thus important to classify terminal cells objectively. To this end, we analyzed the recently published single-cell expression levels of 93 genes in 363 cells of the L1 stage larvae of *C. elegans* [28]. Three of the 363 cells are not terminal cells in the lineage considered here and are thus removed. We then classified the remaining 360 terminal cells into eight types (**Fig. S5I**) based on transcriptome similarities among cells (see Materials and Methods), because the terminal cells of the *C. elegans* lineage analyzed here were previously classified into eight functional types after the removal of apoptotic cells [26], [29]. Although the new classification differs substantially from the previous classification (**Fig. S5I**), the rare-early correlation in the *C. elegans* lineage remains highly significant under the new classification (*P*<0.0001, compared with 10,000 random lineages with the same lineage tree structure; **Fig. S5J**), and this result is insensitive to the number of cell types classified (from 4 to 40) (**Fig. S5K**). Because only 360 of the 671 terminal cells in the *C. elegans* lineage analyzed here have the single-cell gene expression data, the new cell type classification is incomplete and hence cannot be used to calculate *f*_n_ and *f*_p_ (see **Table S1**). But, because cell type reclassification does not alter the shallowness and tree structure of the lineage and because the rare-early correlation clearly remains unchanged, the reclassification should not qualitatively affect *f*_n_ and *f*_p_.

Besides its contribution to robustness, the rare-early correlation has other potential implications. For instance, the risk of mutation is minimized for cells that appear early. In the case of the *C. elegans* cell lineage analyzed here, the early appearance of the two germ cells may have reduced the germline mutation rate per nematode generation. Note, however, that the rare-early correlation is unlikely to have been caused by natural selection for a low germline mutation rate, because the correlation remains strong (ρ = 0.515; *P*<10^−38^) even when the germ cells are not considered. A potential alternative explanation of the rare-early correlation is that the rare cells have physiological roles to support the developing embryo and hence need to be produced earlier. However, this hypothesis does not appear to be empirically supported. For example, the two germ cells that appear very early in the cell lineage have no physiological role in supporting the developing embryo.

### Non-clonality of cell types contributes to robustness

Intuitively, one may think that cell types are clonal, meaning that all terminal cells of a cell type form a monophyletic or paraphyletic group in a cell lineage tree [32], [33]. This intuition, however, is incorrect. Studies in multiple animal species have shown that cell types are typically nonclonal or polyphyletic [34]–[41], meaning that the terminal cells of the same cell type are derived from multiple sublineages (e.g., see **Fig. 1A**). If we compare two cell lineages with the same tree structure, an internal cell death will kill the same number of terminal cells in the two lineages, but the dead terminal cells are more likely to be of the same type in the clonal lineage than in the non-clonal lineage. Because the loss of multiple terminal cells of the same type tends to result in a lower *f* than the loss of the same number of terminal cells distributed among several types (see Materials and Methods), we predict that clonality reduces lineage robustness while non-clonality improves lineage robustness.

To test the above hypothesis, let us measure the clonality of a cell lineage by
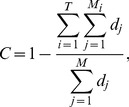
(2)where *T* is the total number of terminal cell types, *M* represents all pairs of terminal cells, where a subscription of *i* limits the cell pairs within cell type *i*, and *d_j_* is the lineal distance between cell pair *j*, which is the number of edges on the shortest path connecting the cell pair (e.g., the lineal distance between the two T3 cells in **Fig. 1A** is 4). For a given lineage tree structure, 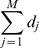 in the equation is fixed, whereas 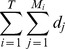 decreases with clonality. Thus, the stronger the clonality, the larger the *C*.

**Figure 5:**
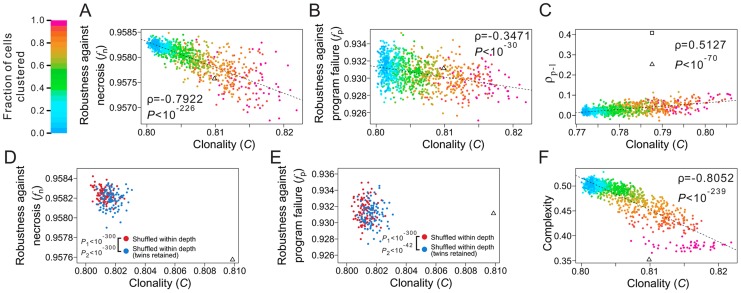
Non-clonality of cell types contributes to the robustness of the *C. elegans* lineage. (A–B) Lineage robustness in the presence of (A) necrosis or (B) program failure declines with the rise of clonality in 1050 random lineages with different levels of clonality. These lineages are generated by different degrees of clustering of terminal cells of the same types while constraining the lineal topology and depths of all terminal cells as in *C. elegans*. The different degrees of clustering are shown by different colors, with the scale shown at the top left corner of the figure. The dashed line is the linear least-square regression. The rank correlation between clonality and robustness, as well as the associated *P*-value, are presented. The *C. elegans* lineage is indicated by a triangle. (C) Spatial requirements cannot explain the low clonality of the real lineage. The 1050 random lineages with different levels of clonality are plotted, showing that the increase of clonality promotes the rank correlation (ρ_p-l_) between *p*hysical and *l*ineal distances of terminal cells of the same type. The triangle indicates the real lineage, while the square indicates an artificial lineage with the same topology, depths of all cells, and clonality as the real lineage, but a higher ρ_p-l_. The *C* values here are different from those appearing in panels A, B and F, because not all *C. elegans* terminal cells have three-dimensional coordinates. (D–E) Spatial constraint lowers lineage robustness in the presence of (D) necrosis or (E) program failure. Red dots are random lineages generated by random rearrangement of terminal cells within their respective depths, whereas blue dots are generated with the additional constraint that twin terminal cells, which share their immediate progenitor and their cell type, are maintained. *P*_1_ is the probability that the clonality is equal between the blue and red dots (Mann-Whitney *U* test), whereas *P*_2_ is the probability that the robustness is equal between the blue and red dots (Mann-Whitney *U* test). All *P* values are calculated based on 10,000 red and 10,000 blue dots. For clarity, only 100 red and 100 blue dots are shown here. (F) Lineage complexity decreases with the rise of clonality among the 1050 random lineages. The rank correlation between clonality and complexity, as well as the associated *P*-value, are presented. The *C. elegans* lineage is indicated by a triangle.

To examine the role of clonality on robustness, we controlled all known lineage features that contribute to robustness. Specifically, from a real cell lineage, we rearranged the terminal cells without altering their respective depths and generated a series of random lineages with different levels of clonality (see Materials and Methods). We found that (i) *C* is lower in the three real lineages (triangles in **Fig. 5A**; **Figs. S6A, B**) than in the corresponding random lineages where all cell types are as clonal as possible given the constraints of the lineage tree structure and cell depths (purple dots; *P*<0.12, 0.02, and 0.02, respectively) and (ii) a decrease in *C* leads to an increase in lineage robustness to both necrosis (**Fig. 5A**; **Figs. S6A, B**) and program failure (**Fig. 5B**; **Figs. S6C, D**). These observations support our hypothesis that the non-clonality of the real lineages improves their robustness. Notably, the non-clonality, in combination with the previously identified lineage features, seems sufficient to explain the observed robustness of the real lineages (**Fig. 5A, B**).

A potential alternative explanation of the non-clonality of the real lineages is that it is dictated by some spatial requirements for terminal cells. Because not all cells of the same type are physically proximate (e.g., epithelial cells on the two hands of a person) and because cells may possess limited abilities to migrate, be costly to migrate, and/or have reduced migration under natural selection for rapid development [24], [42], some cell types are necessarily non-clonal. To examine whether some spatial requirements have dictated the reduction of the clonality of the real lineages and as a result created the observed robustness as a byproduct, we obtained the three-dimensional spatial coordinates of terminal cells in *C. elegans* [43]. We then calculated Spearman's rank correlation (ρ_p-l_) between the *p*hysical distance and *l*ineal distance for all pairs of terminal cells that belong to the same type (see Materials and Methods). The *C. elegans* lineage indeed has a much greater ρ_p-l_ (triangle in **Fig. 5C**) when compared with the above generated random lineages of similar *C* (dots in **Fig. 5C**). Nevertheless, the *C. elegans* ρ_p-l_ is apparently not maximized, because we were able to acquire an even higher ρ_p-l_ by rearranging the terminal cells of the *C. elegans* lineage within their respective depths (square in **Fig. 5C**). More importantly, a comparison among the above generated random lineages shows that ρ_p-l_ tends to increase with *C*, suggesting that lowering *C* does not help raise ρ_p-l_. In other words, the observed low clonality cannot be explained by the spatial requirements.

Notwithstanding, the *C* value of each real lineage remains significantly greater than that when all of its terminal cells are completely randomly situated within their respective depths (dark blue dots in **Fig. 5A**; **Figs. S6A–B**; *P*<0.02 in all three species). Two factors may have constrained the further reduction of *C* and the further rise of robustness in the real lineages. The first potential constraint arises from a demand for spatial proximity of certain terminal cells of the same type. Because cell migration is limited [24], [42] and because some cell types exert their functions through physical connections such as in the neural system and muscles, there is a requirement for a large number of terminal cells of the same type to be produced next to one another, which hinders a further reduction of *C*. To test this hypothesis, we focused on pairs of terminal cells that share their immediate progenitor as well as their cell type. By analyzing the spatial coordinates of terminal cells, we found that such “twin” terminal cells are significantly closer physically to each other than each is to other cells of the same type (*Z* = −15.32, *P*<10^−52^; see Materials and Methods), suggesting that the existence of such “twin” terminal cells is in part a result of the spatial requirements. There are also significantly (*P*<10^−4^) more twin terminal cells in *C. elegans* (155) than the random expectation (59.4), which is estimated by randomly rearranging terminal cells within their respective depths. To examine if this spatial requirement constrains the reduction of clonality and the rise of robustness, we retained the relationship of twins while randomly rearranging the terminal cells within their respective depths. In support of our hypothesis, a greater *C* and smaller *f*_n_ and *f*_p_ are observed after this type of rearrangement (blue dots in **Figs. 5D, E** and **Figs. S6E–H**), compared with the rearrangement without the twin constraint (red dots).

The second potential constraint is lineage complexity [26], which is the number of division programs used in the lineage, as shown in **Fig. 1B**. Azevedo and colleagues suggested that lineage complexity has been selectively minimized in evolution [26]. Because lineage complexity negatively correlates with *C* (**Fig. 5F**; **Figs. S6I, J**), selection for lower complexity is expected to increase clonality and hence decrease lineage robustness.

Taken together, our analyses revealed lower-than-expected clonalities in the real cell lineages, which have likely resulted from the potential selection for lineage robustness. Why the clonalities are not further reduced may be explained by the spatial constraints of certain cells and the possible selection for lineage simplicity.

### Natural selection can generate the observed robustness of developmental cell lineages

We have demonstrated the robustness of the three animal cell lineages to necrosis and program failure and have identified a number of lineage features or mechanisms that underlie the observed robustness. But how did such lineage robustness originate? It is not obvious that natural selection for high robustness during the evolutionary expansion of cell lineages will result in high robustness, because today's lineage is greatly restricted by its ancestral forms. For instance, 577 terminal cells have the same fate in the lineages of the two nematodes *C. elegans* (671 terminal cells) and *P. marina* (638 terminal cells) [26]. Below we investigate by computer simulation if the observed robustness of the three cell lineages is achievable simply by adaptation to necrosis or program failure during the evolutionary expansion of cell lineages.

Our simulation, named “macroevolution”, mimics the expansion of a cell lineage in macroevolution by stepwise additions of new terminal cells via divisions of existing terminal cells. That is, upon a division, one daughter cell inherits the identity of its parental cell while the other evolves into a new cell type (see Materials and Methods). Our simulated lineages are the same as the real lineages in the number and identities of terminal cells, but differ in lineal topology and terminal cell depths. Different intensities of selection for high *f*_n_ or high *f*_p_ are applied during the course of the macroevolution. The actual evolution of developmental cell lineages does not necessarily proceed as our macroevolution simulation, because cell fate may change in evolution [24]. In other words, the actual evolution of developmental cell lineages may be more flexible and our overly-constrained macroevolution likely reveals the lower limit of robustness achievable under natural selection for robustness.

**Figure 6:**
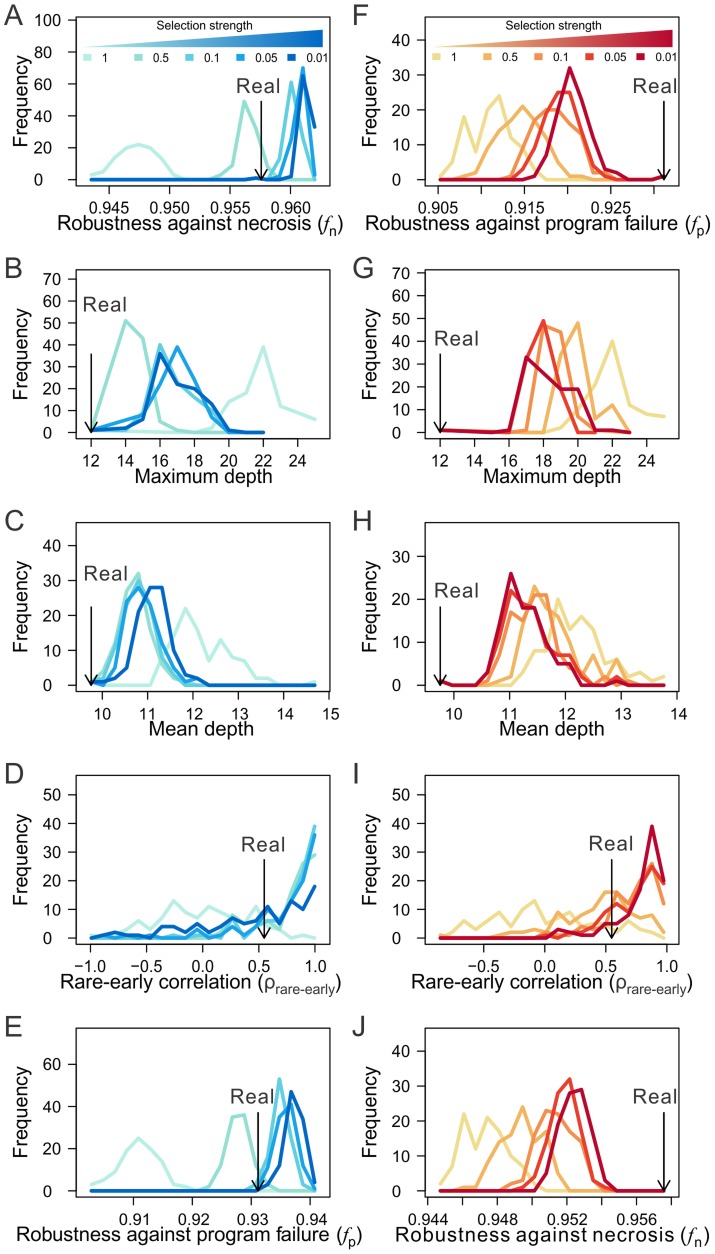
The macroevolution simulation suggests the possibility that the robustness of the *C. elegans* lineage arose as an adaptation to necrosis but not program failure. (A–E) Frequency distributions of (A) lineage robustness in the presence of necrosis (*f*_n_), (B) maximum depth, (C) mean depth, (D) rare-early correlation, and (E) lineage robustness in the presence of program failure (*f*_p_) among lineages generated from the macroevolution with different intensities of selection for high *f*_n_. The observed values from the *C. elegans* lineage are indicated by black arrows. Each distribution in each panel is based on 100 simulation replications. The number next to the color scheme shows the fraction of most robust lineages from which the progenitor of next evolutionary expansion of cell lineage is randomly chosen. That is, the lower the number, the stronger the selection. (F–J) Frequency distributions of (F) lineage robustness in the presence of program failure (*f*_p_), (G) maximum depth, (H) mean depth, (I) rare-early correlation, and (J) lineage robustness in the presence of necrosis (*f*_n_) among lineages generated from the macroevolution with different intensities of selection for high *f*_p_. The observed values from the *C. elegans* lineage are indicated by black arrows.

We found that selection for high *f*_n_ can result in lineages that have similar levels of *f*_n_ as observed in the real lineages (when the selection intensity is 0.5), while removing the selection results in lineages of much lower *f*_n_ (**Fig. 6A**; **Figs. S7A, K**). Compared with the lineages generated under no selection, those generated by selecting for high *f*_n_ exhibit the features known to improve *f*_n_, including the lineage shallowness (although slightly less extreme than in the real lineages) and rare-early correlation (**Figs. 6B**–**D**; **Figs. S7B–D, L–N**). Clonality *C* is not compared because it is not comparable among lineages with different tree structures. Intriguingly, selection for high *f*_n_ also increases *f*_p_ (**Fig. 6E**; **Figs. S7E, O**). On the contrary, selection for high *f*_p_ fails to recapitulate the observed level of *f*_p_ or *f*_n_ (**Figs. 6F, J**; **Figs. S7F, J**), except in the case of the *H. roretzi* lineage (**Figs. S7P, T**), which may be too small to be informative. While selection for high *f*_p_ does result in shifts of the robustness-enhancing lineage features in the predicted directions, these shifts tend not to reach the levels observed in the real lineages (**Figs. 6G**–**I**; **Figs. S7G–I, Q–S**; see Discussion). These findings reveal the possibility that the higher-than-expected *f*_n_ and *f*_p_ in the real lineages are both due to natural selection for high *f*_n_. In other words, the observed high *f*_p_ in the real lineages is probably a byproduct of the selection for high *f*_n_. Consistent with this conclusion is the finding that *f*_n_ and *f*_p_ are highly positively correlated among various randomized lineages generated from the real lineages (**Figs. S7U–W**). While our simulation does not prove that the higher-than-expected *f*_n_ and *f*_p_ in the three animal cell lineages are caused by selection for *f*_n_, it does provide strong evidence for the viability of the hypothesis.

### Lineage robustness is unexplainable by natural selection for lineage simplicity

**Figure 7:**
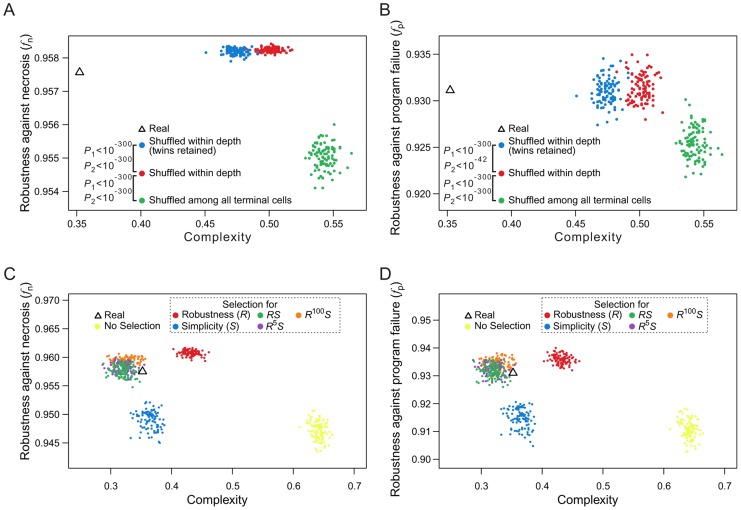
Selection for simplicity cannot explain the robustness of the *C. elegans* cell lineage. (A–B) Complex relationships between lineage complexity and robustness to (A) necrosis or (B) program failure among three types of random lineages. Each dot represents a random lineage, whereas the triangle shows the real lineage of *C. elegans*. *P*_1_ is the probability that the complexity is equal between the dots of two colors compared (Mann-Whitney *U* test), whereas *P*_2_ is the probability that the robustness (*f*_n_ or *f*_p_) is equal between the dots of two colors compared (Mann-Whitney *U* test). All *P* values are calculated based on 10,000 dots of each color. For clarity, however, only 100 dots of each color are shown here. (C–D) Lineage complexity and robustness to (C) necrosis or (D) program failure of lineages generated in the macroevolution. Each evolutionary simulation is conducted 100 times, shown by 100 dots of the same color. The quantity being selected for is defined in the symbol legend, where *S* and *R* represent simplicity (i.e., 1/complexity) and robustness against necrosis (*f*_n_), respectively. The fitness functions used in various simulations (see Materials and Methods) are shown in the dash-lined box. The actual *C. elegans* lineage is indicated by a triangle.

When rearranging terminal cells of a real lineage within their respective depths, we showed that increasing lineage clonality reduces lineage robustness (**Figs. 5A, B**) and complexity (**Fig. 5E**), implying a positive correlation between robustness and complexity. A more extensive analysis, however, revealed that the correlation between robustness and complexity may be positive or negative, depending on the groups of random lineages compared (**Figs. 7A, B**). For example, comparing lineages generated by shuffling terminal cells within their respective depths (red dots in **Figs. 7A, B** and **Figs. S8A–D**) and those generated by completely shuffling all terminal cells (green dots), we found that the rare-early correlation increases robustness but decreases complexity. By contrast, when we compare the lineages generated by within-depth shuffling with (blue dots in **Figs. 7A, B** and **Figs. S8A–D**) or without (red dots) retaining twin terminal cells, we found a positive correlation between robustness and complexity, suggesting that the spatial constraint of terminal cells decreases complexity at the cost of robustness.

Because lineage robustness and complexity are sometimes negatively correlated, selection against complexity (or for simplicity) [26] may result in high robustness, and vice versa. We thus used our macroevolution simulation with relatively strong selection (intensity = 0.05) to investigate if selection for robustness to necrosis or simplicity alone can account for both the robustness and simplicity of the real lineages. To investigate the interplay between robustness and simplicity, we used different fitness functions in the macroevolution simulation with different weights for robustness (R) and simplicity (S) (dash-lined box in **Figs. 7C, D**; **Figs. S8E–H**, where the relative weights for R and S are reflected by the power of R; see Materials and Methods for details). Four observations were made (**Figs. 7C, D**; **Figs. S8E–H**). First, selection for simplicity alone enhances simplicity but not robustness (*f*_n_ or *f*_p_), suggesting that robustness cannot be a byproduct of selection for simplicity (blue dots in the figures). Second, selection for robustness to necrosis alone enhances robustness (*f*_n_ and *f*_p_) as well as simplicity (red dots). Nevertheless, the resulting lineages are still more complex than the real lineages, suggesting that the simplicity of the real lineages may be partially but not entirely due to selection for robustness to necrosis. Third, simultaneous selections for both robustness and simplicity can generate lineages that are similar to the real lineages in *f*_n_, *f*_p_, and simplicity (green, purple, and orange dots), supporting the actions of both selective forces in the evolution of the real lineages. Fourth, under the parameters used, selection for robustness alone generates lineages that exceed the real lineages in *f*_n_ and *f*_p_, whereas this disparity disappears under simultaneous selections for robustness and simplicity, supporting the notion that a requirement for simplicity prevents a further increase in robustness.

## Discussion

### Determinative developmental cell lineages are robust to cell deaths

Phenotypic robustness is an important characteristic of life, but its underlying mechanisms and evolutionary origins are not well understood. In this article, we demonstrated that determinative developmental cell lineages are robust to necrosis and cell division program failure, which approximately represent environmental (or somatic) and genetic (i.e., germline) disturbances, respectively. Although some germline mutations with incomplete penetrance may act like environmental perturbations and some environmental perturbations impact specific programs and hence behave like germline mutations, our analyses included both types of disturbances. We further showed by computer simulation that such robustness can arise from natural selection for robustness against necrosis during the evolutionary expansion of cell lineages. Our findings thus reveal a new mechanism of phenotypic robustness as well as its potential evolutionary origin. Different from almost all previously studied mechanisms of phenotypic robustness, which act at the subcellular or cellular levels, cell lineage robustness manifests at the supracellular level and hence is a unique feature of multicellular organisms. Our study also provides a novel explanation of the contribution of ontogenesis to phenotypic robustness.

Necrosis caused by environmental stresses or somatic mutations are unavoidable [27]. Similarly, cell division program failure is expected to occur occasionally due to germline mutations [27]. Although mechanisms buffering stresses and mutations may exist such that the rate of necrosis or division program failure is lowered [2]–[5], [44], cell lineage robustness is likely an important mechanism buffering the adverse effect of necrosis and program failure upon their occurrence. This mechanism is especially important for species whose development is predominantly determinative because the cell fate is largely fixed in these organisms. Cell lineage robustness, along with regulative development (see below), complements subcellular and cellular mechanisms of robustness to form a multi-layer defense system against environmental and genetic perturbations that are common in nature.

A highly related subject is the robustness of sublineages, which are lineages starting from non-zygote cells. Although our definition of robustness is directly applicable to sublineages, the expectation for sublineage robustness may vary, depending on the specific types of sublineages. For instance, regulative development occurs to a small number of cells during the late development of *C. elegans*, where cell losses may sometimes be compensated by additional divisions of neighboring cells [25]. The sublineages with the ability of such compensatory growth, conferred by regulative development, may not have the typical properties of robust lineages such as low cell depths. Compensatory growth may be further generalized to include sublineages that are populated by stem cells, commonly seen in arthropods and vertebrates [45]. Other than delaying the development, such compensatory growth can apparently solve the problems caused by necrosis [46]. The existence of compensatory growth demonstrates canalization mechanisms other than the cell lineage robustness revealed here, further supporting the importance of having a robustness developmental process. With a modification of our model, stem cells may be included such that the robustness of developmental cell lineages can be evaluated for organisms with prevalent compensatory growth.

Our findings of cell lineage robustness and its mechanisms offer important biological insights. For example, somatic-mutation-based analysis in mice revealed that cells of the same type from the same organ such as the kidney have several different lineal histories [38], [39]. We showed that this phenomenon of non-clonality is beneficial to lineage robustness, at the cost of lineage simplicity. Thus, the benefit of having increased robustness may exceed the cost of having extra division programs for these cell types. In general, features contributing to lineage robustness (e.g., rare cells are produced relatively early in ontogeny) may be found to be detrimental to other traits (e.g., common cells would have relatively high probabilities of death). Knowing the underlying tradeoffs helps understand the origins of such detrimental features, which may lead to potential solutions.

### Evolutionary origins of cell lineage robustness

One intriguing finding from our macroevolution simulation is that both *f*_n_ and *f*_p_ can be raised by natural selection for high *f*_n_ but not as effectively by selection for high *f*_p_. This disparity is not because the parameters we used render the cell death rate higher in the presence of necrosis than in the presence of program failure. In fact, the opposite is true (e.g., see **Fig. 1E–J**). The disparity may be related to the larger variation in *f*_p_ than *f*_n_ among individuals whose expected lineages under no cell death are identical. Even when the rates of necrosis and program failure are fixed, individuals with the same expected lineages can still have different *f* values because necrosis and program failure are stochastic. Based on the necrosis and program failure rates used here, we estimated in *C. elegans* that the standard deviation of *f*_n_ among individuals is 0.1095, while that of *f*_p_ is 0.1745. It is reasonable to assume that a larger variation in *f* translates into a larger variation in fitness. Because the larger the variation in fitness among (isogenic) individuals, the lower the efficacy of natural selection [47], selection for *f*_p_ is less effective than selection for *f*_n_ in raising lineage robustness. Nevertheless, whether the standard deviations of *f*_n_ and *f*_p_ are directly comparable is unclear and other explanations may exist.

Our finding that cell lineage robustness can result from natural selection for robustness, coupled with previous findings on the possibility of selection for genetic and environmental robustness [2], [3], [7], [48], suggests the likelihood that the observed cell lineage robustness against necrosis and program failure is a direct result of natural selection for robustness. Nevertheless, we cannot exclude the possibility that the observed robustness results partially or entirely as a byproduct of other selections or generative biases [49]. For example, we showed that a low maximum depth improves the robustness of a cell lineage and that the maximum depths of the three animal cell lineages are identical or close to their theoretical minimums. Although selection for robustness against necrosis may explain this phenomenon (**Fig. 6B**), selection for short developmental time is another possible explanation [50]. It was previously thought that the robustness-enhancing feature of non-clonality of the cell lineages of *C. elegans* and *P. marina* results from selection for rapid development that avoids the time-consuming cell migration [24]. But the recently determined nearly complete cell lineages of two other nematodes that develop slowly (*Halicephalobus gingivalis* and *Rhabditophanes* sp.) exhibit similar non-clonality [51], [52], suggesting that selection for rapid development is not the cause of the observed non-clonality in nematodes. Furthermore, selection for fast development cannot explain other robustness-enhancing features of cell lineages (e.g., early appearances of rare cell types). Given the presence of multiple robustness-enhancing features in animal developmental cell lineages, natural selection for robustness is a plausible and the most parsimonious explanation of the origin of lineage robustness. The generative bias hypothesis would assert that the observed high robustness of developmental cell lineages is caused by mutational biases. But it is difficult to imagine that such biases would generate multiple nonrandom features that all happen to increase lineage robustness.

### Limitations and future improvements

Our estimation of cell lineage robustness is based on several assumptions. First, we used a necrosis rate of one cell per lineage and a program failure rate of 1/*N*_internal_ per program, where *N*_internal_ is the total number of internal cells in the lineage. These parameters are probably lower than the actual rates, rendering our estimates of cell lineage robustness conservative. Our results should be verified in the future with the real necrosis and program failure rates when they become available. It is also possible that the rate of necrosis and that of program failure vary among cells and programs [53]. Such information, when it becomes available, can be incorporated into our model (**Fig. S1K, L**) to achieve a more accurate estimate of cell lineage robustness.

Second, the robustness function, as described by Eq. (1) under *a_i_* = 1, may not be accurate, because of (i) variable importance of cells of different types, (ii) imprecise cell type classification, (iii) interactions among different cell types, and/or (iv) potential compensation of cell death by other mechanisms. Yet, our conclusion appears robust to variable impacts of different cell types, because the use of different *a_i_* values yielded similar results (**Fig. S1A–F**). Our conclusion is also highly robust to cell type classification, because transcriptome-based and function-based classifications yielded similar results that are invariant to the number of cell types classified (**Figs. S5I–K**). Further division of a cell type into subtypes did not alter our results (**Fig. S1G, H**). Our results also appear to be robust to the variation in the necrosis rate among cells or the failure rate among programs (**Figs. S1K–L**). While necrotic cell death is rarely compensated by regeneration of the corresponding live cells in *C. elegans* [25], this may not be the case in some complex organisms especially during late development [46]. Similarly, it is possible that only some but not all descendants of an affected internal cell get lost. By contrast, when cell induction or replacement occurs in cell fate determination, a cell death may lead to the loss of terminal cells that are non-descendants of the dead cell. Furthermore, a perturbation may induce the production of extra terminal cells (e.g., by blocking apoptosis [54]). These variations, as well as potential interactions among different cell types, have not been considered in our analyses, but can be studied in the framework developed here when detailed information about these processes becomes available.

Third, it is possible that the potential type of an internal cell (i.e., its division program) is limited genetically, but such a constraint is not explicitly modeled in our randomization of cell lineages. With a better understanding of this constraint, we can refine our randomization in the future. Nonetheless, it is worth mentioning that the expression profiles of 93 genes examined in *C. elegans* terminal cells were found to be largely determine by their cell types [28], suggesting that the division programs of internal cells are not so limited, because otherwise the transcriptomes of the terminal cells should be dictated by their lineal histories.

By progressively constraining various features of a cell lineage, we identified several contributors to lineage robustness, including terminal cell depths, lineal topology, early appearances of rare cells, and non-clonality of cell types. Although the impacts of any two of these characteristics to lineage robustness are not completely overlapping, it is important to note that they are not completely non-overlapping either. As such, it is difficult to assess the relative contributions of these characteristics to lineage robustness. Although all of these characteristics exist in the three animal cell lineages examined, variations are expected when additional species are examined. For instance, in most organisms, primordial germ cells are set aside early in development [55]. In mice, however, these cells appear at a much later stage [40], [56], which likely reduces the rare-early correlation and the cell lineage robustness. But, mouse blastomeres up to the eight cell stage are equipotent [57], which reduces clonality and increases lineage robustness.

Using the macroevolution simulation, we showed that, in the evolutionary expansion of cell lineages, adaptation to random necrosis can result in highly robust cell lineages. Our simulation is a coarse-grained approximation rather than a precise description of the evolution of developmental cell lineages. However, because our simulation explicitly models historical contingency, the constraint imposed by ancestral lineages on future lineages, our simulation is more realistic than that by swapping sublineages in a real or random lineage [26]. Our model can be further improved by including the genetic networks that underlie cell fate determination [49] when such information becomes available. It can also be improved by allowing lineages to expand through divisions of internal cells rather than only terminal cells.

### Outlook

Decoding the developmental cell lineages of the human and other organisms is a grand scientific challenge (http://www.lineage-flagship.eu/). With the rapid advancement of genomics [58], especially single-cell genome sequencing [59]–[61], it will not be long before one can use somatic mutations accumulated during ontogenesis to reconstruct the cell lineages of complex organisms such as mammals [62], [63]. Our computational analysis of the three determinative cell lineages provides experimentally testable [39] hypotheses on the organizing principles of developmental cell lineages and opens the door toward characterizing systemic properties of complex cell lineages, an area that promises to be of both theoretical and applied values in understanding evolution, development, and carcinogenesis.

## Materials and Methods

### Developmental cell lineages

To reliably evaluate lineage robustness as defined in Eq. (1) of the main text, the cell lineage data must satisfy the following four criteria (**Table S1**). First, the lineage has the form of a binary tree. Second, the lineage starts from the zygote and contains all cells up to a developmental stage with at least 100 cells. Third, all the terminal cells at this stage must be included in the lineage data. Fourth, the terminal cells should be functionally categorized because the impact of a cell death depends on the cell type. There are only three developmental cell lineages that meet all these criteria (*C. elegans*, *P. marina*, and *H. roretzi*) and they were retrieved from an earlier publication [26]. Several other well-known cell lineages do not satisfy one or more of the four requirements and thus cannot be used here (**Table S2**). In the *C. elegans* cell lineage, 671 terminal cells were categorized by standard anatomical descriptions [25] as: 39 blast, 113 death, 93 epithelial (arcade, hypodermis, pharyngeal structural, rectum, valves), 2 germ, 13 gland (coelomocytes, excretory system, and pharyngeal glands), 20 intestinal, 123 muscle (including the head mesodermal cell), 46 neural structural cells, and 222 neurons. To consider the potentially different importance of cells of the same type, we subdivided neurons into two excretory canal neurons, 95 interneurons, 45 motor neurons, 26 polymodal neurons, and 54 sensory neurons [26]. We also validated our primary result using an expanded post-embryonic hermaphroditic *C. elegans* cell lineage with a total of 937 terminal cells, including 5 blast, 131 death, 262 epithelial (arcade, hypodermis, pharyngeal structural, rectum, valves), 2 germ, 13 gland (coelomocytes, excretory system, and pharyngeal glands), 20 intestinal, 153 muscle (including the head mesodermal cell), 46 neural structural, and 305 neuronal cells [29], [64]. For *P. marina*, the cell lineage up to muscle contraction, containing 638 terminal cells, were classified as: 81 body muscle, 67 death, 2 germ, 131 hypodermis, 20 intestine, 195 nervous system, 112 pharynx, and 30 unknown fate [24]. For *H. roretzi*, the cell lineage up to the 110-cell stage was used. The terminal cells were classified according to the fates of their descendants [30] as: 12 endoderm, 50 epidermis, 6 mesenchyme (and trunk lateral cells), 10 muscle (and trunk ventral cells), 16 nervous system (brain, nerve cord, palps, primordial pharynx, and sensory pigment cells), 10 notochord, and 6 undifferentiated.

### Estimation of *f*_p_

If the program failure rate is *p*, the number of programs that fail in a lineage is a random variable *b* following the binomial distribution B(*N*_program_, *p*), where *N*_program_ is the total number of unique programs in the lineage. After randomly picking *b* failed programs, we calculated *f* using Eq. (1). The above step was repeated 10*N*_all_ times to calculate the expected *f*. Here *N*_all_ is the total number of cells in the lineage. We used *p* = 1/*N*_internal_, where *N*_internal_ is the number of internal cells in the lineage. The above assumed relationship between *p* and *N*_internal_ ensures that the expected number of internal cells whose division programs fail is the same between a real lineage and all of its randomized lineages, which is required for a fair comparison of their *f*_p_ values. For two reasons, the stochasticity involved in the estimation of *f*_p_ is unavoidable. First, despite the constancy in the expected number of cells with failed programs, the expected number of failed programs varies between a real lineage and its random lineages. Second, because *b* could be large, it is computationally impossible to explore all possibilities in the event of multiple program failures.

### Generation of random cell lineages

We generated random lineages under eight different constraints. (i) We randomly coalesced the terminal cells of a real lineage (**Figs. 1E**–**J**) using the following procedure. Suppose there are *m* terminal cells. We randomly pick two of them (regardless of their cell types) and coalesce them, meaning that they become sisters and share the immediate progenitor cell. There are now *m*-1 cells left (*m*-2 terminal cells and 1 progenitor cell). We then randomly pick two cells from these *m*-1 cells and repeat the coalescence process until there is only one cell left. This process generates a random cell lineage of the *m* terminal cells (**Fig. S2**). (ii) We constrained the random coalescent process such that the maximum depth is fixed at a predetermined value (**Figs. 2B**–**D**). (iii) We constrained the random coalescent process such that the maximum depth is fixed at a predetermined value and the mean depth is close to a predetermined value (i.e., cell depths in a random lineage is a bootstrap sample of the real depths) (**Figs. 2E, F**). (iv) We generated random lineages by constraining the distribution of the depths of all terminal cells as in a real lineage but allowing variation of the lineage tree structure and depths of individual cells. In procedures (iii) and (iv), when the set of cell depths is given, each terminal cell is randomly assigned with one of the depths. We then randomly paired-up the *x_y_* cells at the maximum depth *y* as sister cells, creating *x_y_*/2 internal cells at depth *y*-1. It is repeated at depth *y*-1 for the (*x_y_*/2+*x_y_*_-1_) cells, and then recursively at depth *y*-2, *y*-3, …, and 1 (**Figs. 3A, B**). (v) We generated random lineages that have the same topology as a real lineage and then randomly shuffled all the terminal cells (**Figs. 3C, D**; **Figs. 4B**–**D**; **Figs. 7A, B**). (vi) We shuffled all the terminal cells in a real lineage within their respective depths (**Figs. 5D, E**; **Figs. 7A, B**). (vii) In addition to the constraint in (vi), we further maintained the twin terminal cells as twins in shuffling (**Figs. 5D, E**; **Figs. 7A, B**). (viii) We first defined a random order of cell types. Within each depth, *g* percent of terminal cells in every type are picked and sorted by the predefined type order, while the remaining unsorted cells are randomly inserted into the sorted list of cells. The cells are then assigned in the order of appearance in the list to the terminal nodes at that given depth from the left to the right of the lineage. The procedure is repeated for every depth (with the same cell type order) to create a lineage whose clonality increases with *g* (**Figs. 5A–C, F**). For all except the second and eighth constraints, we generated 10,000 random lineages. For the second constraint, we set the maximum depth as small as *D*_min_ and as large as *D*_real_+2(*D*_real_−*D*_min_+1), where *D*_min_ is the theoretical lower limit of the lineage's maximum depth and *D*_real_ is the observed maximum depth of the real lineage. We then generated 5,000 random lineages for each possible maximum depth between these two extremes. For the eighth constraint, we used *g* at every 5th percentile, and created 50 random lineages for each value of *g*. The source codes for generating the random lineages can be downloaded from http://code.google.com/p/eadlin/downloads/list.

### Terminal cell type classification based on single-cell gene expression data

Gene expression profiles of 93 genes in 363 cells at the *C. elegans* L1 stage [28] were retrieved. Three of the 363 cells are not terminal cells in the lineage considered here and are thus removed. We then used the *hclust* function in the R package to hierarchically cluster the 360 cells based on the pair-wise Euclidean distances in the expression levels of the 93 genes. The tree is cut at an appropriate height to acquire a designated number of groups of cells (e.g. cutting at the root will result in two groups); these groups are regarded as transcriptome-based cell types.

### Constraints imposed by the spatial organization of terminal cells

The three-dimensional spatial coordinates of 334 terminal cells in the *C. elegans* lineage were retrieved from a recent paper [43]. The physical distance between two cells is the Euclidian distance between the centers of their nuclei [43]. To search for a cell lineage with ρ_p-l_ greater than that (0.2533) observed in *C. elegans*, we randomly generated 100 lineages that differ from the *C. elegans* lineage by only one swap between two terminal cells of the same depth and type. We chose the lineage with the highest ρ_p-l_ among the 100 random lineages, and repeated this process 100 times to obtain a lineage with ρ_p-l_ = 0.4085 (the square in **Fig. 5C**).

Using terminal cells with three-dimensional coordinates, we calculated the mean physical distance between a pair of twin cells in *C. elegans*. We similarly calculated the mean physical distance between a pair of randomly picked terminal cells of the same type for the same number of pairs as twins. We repeated this calculation 100 times to estimate the mean and standard deviation. These values allowed the calculation of a *Z*-score for the observed value from the twins.

### Simulation of macroevolution of developmental cell lineages

The macroevolution simulation is designed to mimic the evolutionary expansion of a cell lineage under the constraint of its ancestral forms. Basically, the evolution is modeled by repeated additions of terminal cells, and each individual addition is called a round of bifurcation. To ensure that the macroevolution generates a cell lineage that is comparable to a given real lineage, we first completely shuffled the terminal cells of the real lineage to obtain a randomized terminal cell sequence. Starting from the first cell in the sequence as the founder cell, a lineage of *m* terminal cells is evolved by *m*-1 rounds of bifurcation. In each round, one random terminal cell from the evolved lineage is chosen and divided into two daughter cells. After the division, one of the daughter cells inherits the cell type of its parental cell, whereas the other is assigned the type of the next cell in the predetermined terminal cell sequence. The parental cell then becomes an internal cell with a division program generating its original cell type and the new cell type. At each step of lineage expansion, 100 random bifurcations are examined. Among them, a random lineage is chosen from the top *k* robust lineages as the starter of the next round of expansion, where *k* is adjusted between 1 and 100 to represent different selection intensities. The smaller the *k* value, the stronger the selection. In Fig. 6 and Fig. S7, the presented selection intensity equals *k*/100; in Fig. 7 and Fig. S8, *k* equals 5. Regardless of the cell lineage size, we used an expected necrosis rate of 1 necrosis per cell lineage or an expected program failure rate of 1/*N*_internal_ failure per program. For the macroevolution involving selection for simplicity *S* ( = 1/complexity), we calculated lineage complexity [26] at every round of bifurcation and combined it with the robustness (*R*) to define the fitness of a lineage. For instance, *R* ^5^ *S* (**Fig. 7B**) means that fitness = *R* ^5^ *S*. Here *R* equals *f*_n_ defined in Eq. (1). Under each parameter set, we repeated the macroevolution 100 times to access variations. For the developmental cell lineage of *H. roretzi*, to retain its fully symmetric feature during macroevolution, bifurcations were carried out in one half of the lineage, but the robustness was calculated after mirroring the half lineage. The source code of the macroevolution simulation can be downloaded from http://code.google.com/p/eadlin/downloads/list.

### Loss of terminal cells distributed among several cell types versus one type

Based on Eq. (1), it is clear that when different cell types have different numbers of (terminal) cells, a cell death that happens to a common cell type would have a smaller effect on *f* than a cell death that happens to a rare cell type. Now let us consider the scenario of *T* terminal cell types, each with exactly *N* terminal cells. Let *h* terminal cells to die, where 1<*h*<*N*. If all dead cells are of the same type, we have 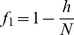. If we arbitrarily assign *h*_1_ (0<*h*_1_<*h*) cell death events to another cell type, we have 
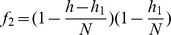
. It can be shown that 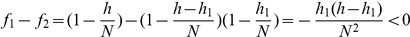. Assigning *h*_1_ cell deaths to a second type and *h*_2_ cell deaths to a third type (0<*h*_2_<*h*-*h*_1_) would result in 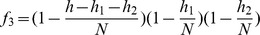. It can also be shown that 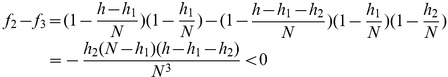. The same is true when the cell deaths are distributed among more cell types. Thus, the loss of multiple terminal cells of the same type tends to result in a lower *f* than the loss of the same number of terminal cells distributed among several types.

## Supporting Information

Figure S1 The robustness of the three animal cell lineages is not sensitive to various simplifying assumptions made in the calculations. (A) Cumulative probability distribution of the relative robustness of the *C. elegans* lineage to necrosis under 1000 sets of *a*_i_ values randomly sampled from a uniform distribution between 1 and 10. Here, the *X*-axis shows the relative robustness, measured by the probability that a random coalescent lineage exceeds the real lineage in *f*_n_ (i.e., equivalent to the *P*-value in Fig. 1E). If one believes that cell types with more cells are physiologically more important than those with fewer cells, *a*_i_ should be positively correlated with *N*_i_. Our conclusions in all panels hold even for the subset of the random lineages in which *a*_i_ is positively correlated with *N*_i_. (B) Cumulative probability distribution of the relative robustness of the *C. elegans* lineage to program failure under 1000 sets of *a*_i_ values randomly sampled from a uniform distribution between 1 and 10. Here, the *X*-axis shows the relative robustness, measured by the probability that a random lineage exceeds the real lineage in *f*_p_, as determined in Fig. 1F. (C) Cumulative probability distribution of the relative robustness of the *P. marina* lineage to necrosis under 1000 sets of *a*_i_ values randomly sampled from a uniform distribution between 1 and 10. (D) Cumulative probability distribution of the relative robustness of the *P. marina* lineage to program failure under 1000 sets of *a*_i_ values randomly sampled from a uniform distribution between 1 and 10. (E) Cumulative probability distribution of the relative robustness of the *H. roretzi* lineage to necrosis under 1000 sets of *a*_i_ values randomly sampled from a uniform distribution between 1 and 10. (F) Cumulative probability distribution of the relative robustness of the *H. roretzi* lineage to program failure under 1000 sets of *a*_i_ values randomly sampled from a uniform distribution between 1 and 10. (G–H) When neurons are divided into subtypes, the *C. elegans* developmental cell lineage is still more robust than its random lineages in the presence of (G) necrosis or (H) program failure. (I–J) The expanded hermaphroditic post-embryonic *C. elegans* developmental cell lineage with 937 terminal cells is more robust than its random lineages in the presence of (I) necrosis or (J) program failure. (K–L) The *C. elegans* developmental cell lineage is more robust than its random lineages in the presence of (K) necrosis or (L) program failure, when the rate of necrosis or program failure varies among cells or programs according to an exponential distribution. In panels G–L, the grey bars show the frequency distribution of the robustness of 10,000 random lineages, whereas the arrow indicates the robustness of the *C. elegans* cell lineage. The random lineages are generated by randomly coalescing the terminal cells of the *C. elegans* lineage. *P*-value indicates the probability that a randomly generated lineage is more robust than the real lineage. *Z*-score is the number of standard deviations (of the random lineages) by which the observation deviates from the random expectation.
(PDF)

Figure S2 Procedure of generating random lineages by the coalescent process.
(PDF)

Figure S3 Low depths of terminal cells improve the robustness of the *P. marina* and *H. roretzi* lineages to necrosis and program failure. (A–L) These panels are the same as in Fig. 2, except for the species examined. In panels (C)–(F) and (I)–(L), the real lineage is indicated by a red triangle for easy recognition.
(PDF)

Figure S4 Lineal topology and terminal cell organization contribute to the robustness of the *P. marina* and *H. roretzi* lineages. (A–H) These panels are the same as in Fig. 3, except for the species examined.
(PDF)

Figure S5 That rare cell types tend to have low depths improves the robustness of cell lineages. (A–H) These panels are the same as in Fig. 4, except that the species examined are *P. marina* and *H. roretzi*. Cell types in panel (A): Ger, germ; Hyp, hypodermis; Int, intestine; Mus, muscle; Ner, nervous system; Pha, pharynx. Cell types in panel (B): End, endoderm; Epi, epidermis; Mes, mesenchyme; Mus, muscle; Ner, nervous system; Not, notochord. (I) Reclassification of the eight *C. elegans* cell types based on expression similarity among cells. The total number of terminal cells belonging to each type is given in the parentheses. For a given functional cell type, the fraction of cells belonging to each expression-based cell type is indicated by the area of the circle in the matrix. The mutual information between the two classifications would be 2.33 if they match perfectly. The actual mutual information is 1.45, indicating a substantial difference between the two classifications. (J) Rare-early correlation in *C. elegans* under the expression-based cell type classification shown in (I). (K) The rare-early correlation in *C. elegans* under the expression-based cell type classification is robust to the number of cell types classified. In each case, the probability that a random lineage has a higher rare-early correlation than that observed in *C. elegans* is smaller than 0.001. The probability is determined as in Fig. 4B.
(PDF)

Figure S6 Non-clonality of cell types contributes to the robustness of *P. marina* and *H. roretzi* cell lineages. (A–J) These panels are the same as panels A, B, D, E, and F in Fig. 5, except for the species examined. There is no data of between-cell physical distances in *P. marina* and *H. roretzi*. Consequently, the analysis in Fig. 5C cannot be conducted for these two species.
(PDF)

Figure S7 The macroevolution simulations for *P. marina* and *H. roretzi* lineages and the correlation between robustness to necrosis (*f*_n_) and that to program failure (*f*_p_). (A–T) These panels are the same as in Fig. 6, except that the species examined are *P. marina* and *H. roretzi*. (U–W) Correlation between *f*_n_ and *f*_p_ among various random lineages generated from the real lineages of (U) *C. elegans*, (V) *P. marina*, or (W) *H. roretzi*. The real lineages are indicated by triangles.
(PDF)

Figure S8 Selection for simplicity cannot explain the robustness of the *P. marina* and *H. roretzi* lineages. (A–H) These panels are the same as in Fig. 7, except for the species examined.
(PDF)

Table S1 Requirements for a developmental cell lineage dataset to be amenable to our analysis.
(PDF)

Table S2 Some well-known developmental cell lineage datasets that are not amenable to our analysis.
(PDF)
