# Supplementary figures and images for: Determinative Developmental Cell Lineages Are Robust to Cell Deaths
Source: PLoS Genet. 2014 Jul 24;10(7):e1004501. doi: 10.1371/journal.pgen.1004501 (PMC4110091; doi:10.1371/journal.pgen.1004501)

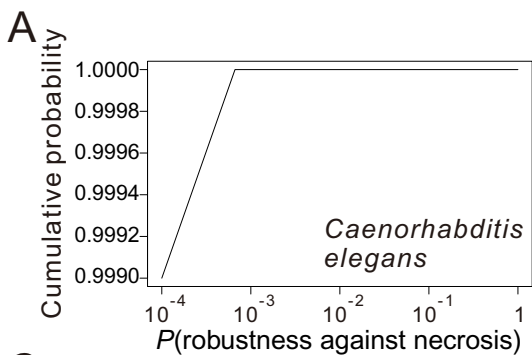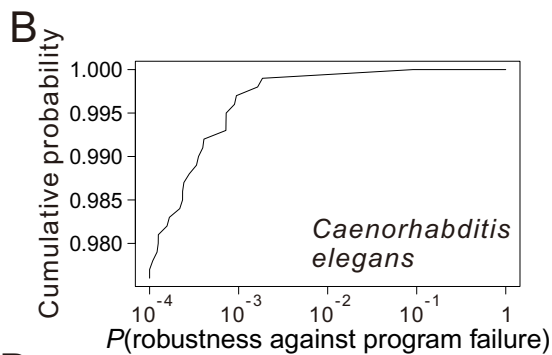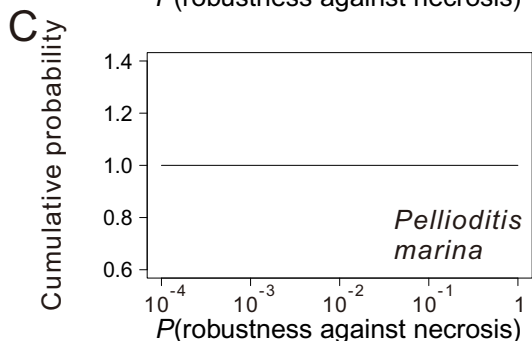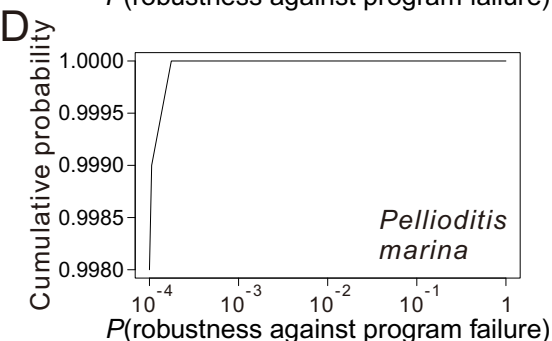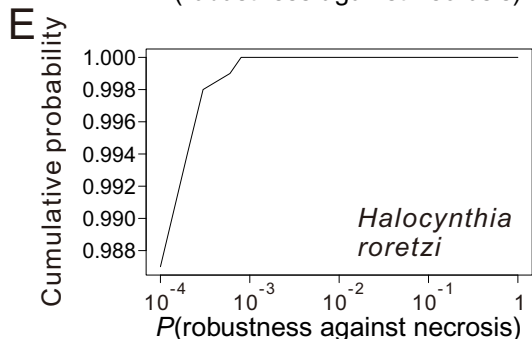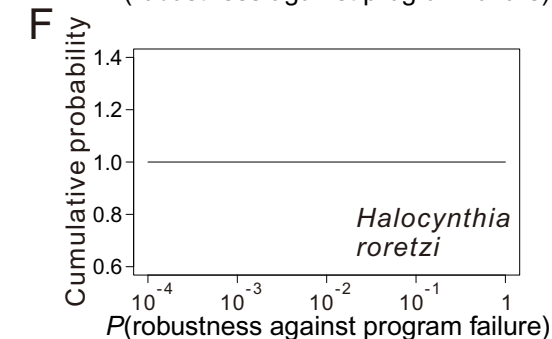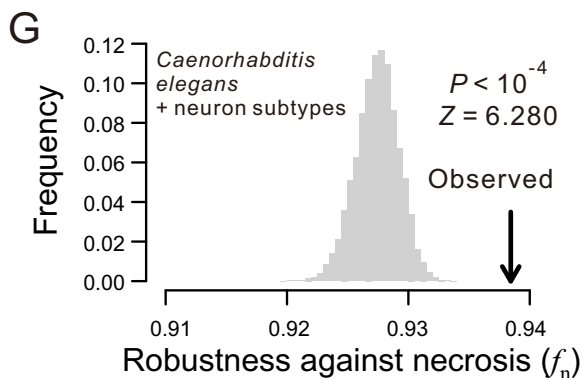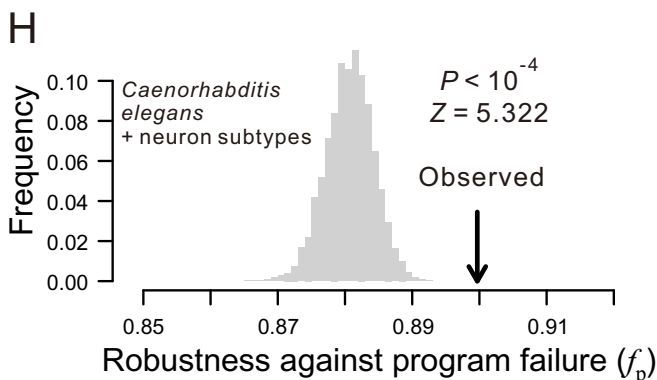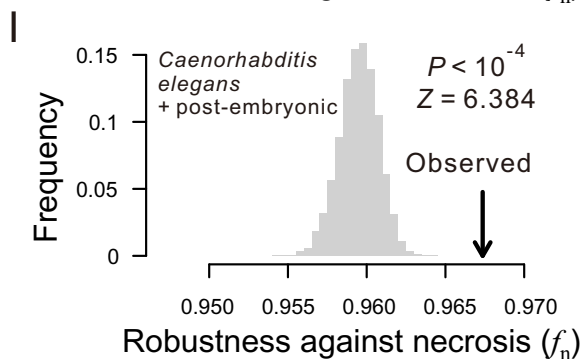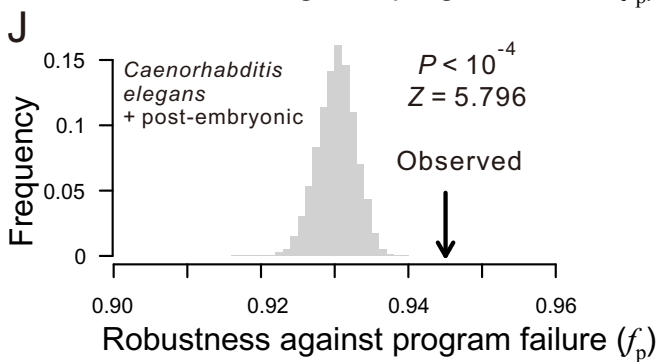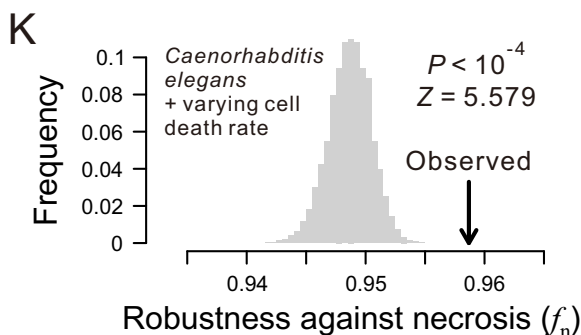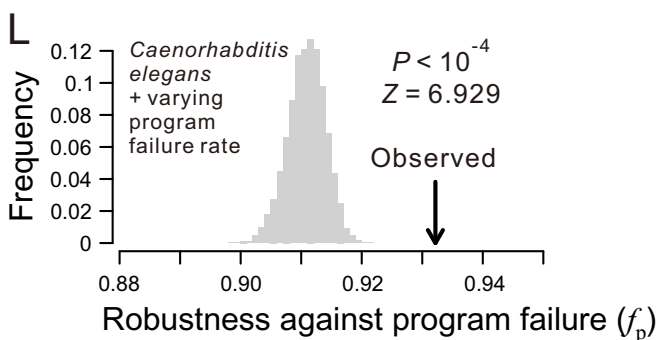

Figure S1

Supplement: Figure S1 — The robustness of the three animal cell lineages is not sensitive to various simplifying assumptions made in the calculations. (A) Cumulative probability distribution of the relative robustness of the C. elegans lineage to necrosis under 1000 sets of a i values randomly sampled from a uniform distribution between 1 and 10. Here, the X-axis shows the relative robustness, measured by the probability that a random coalescent lineage exceeds the real lineage in f n (i.e., equivalent to the P-value in Fig. 1E). If one believes that cell types with more cells are physiologically more important than those with fewer cells, a i should be positively correlated with N i. Our conclusions in all panels hold even for the subset of the random lineages in which a i is positively correlated with N i. (B) Cumulative probability distribution of the relative robustness of the C. elegans lineage to program failure under 1000 sets of a i values randomly sampled from a uniform distribution between 1 and 10. Here, the X-axis shows the relative robustness, measured by the probability that a random lineage exceeds the real lineage in f p, as determined in Fig. 1F. (C) Cumulative probability distribution of the relative robustness of the P. marina lineage to necrosis under 1000 sets of a i values randomly sampled from a uniform distribution between 1 and 10. (D) Cumulative probability distribution of the relative robustness of the P. marina lineage to program failure under 1000 sets of a i values randomly sampled from a uniform distribution between 1 and 10. (E) Cumulative probability distribution of the relative robustness of the H. roretzi lineage to necrosis under 1000 sets of a i values randomly sampled from a uniform distribution between 1 and 10. (F) Cumulative probability distribution of the relative robustness of the H. roretzi lineage to program failure under 1000 sets of a i values randomly sampled from a uniform distribution between 1 and 10. (G–H) When neurons are divided into su [file pgen.1004501.s001.pdf]

*Pellioiditis marina**Halocynthia roretzi*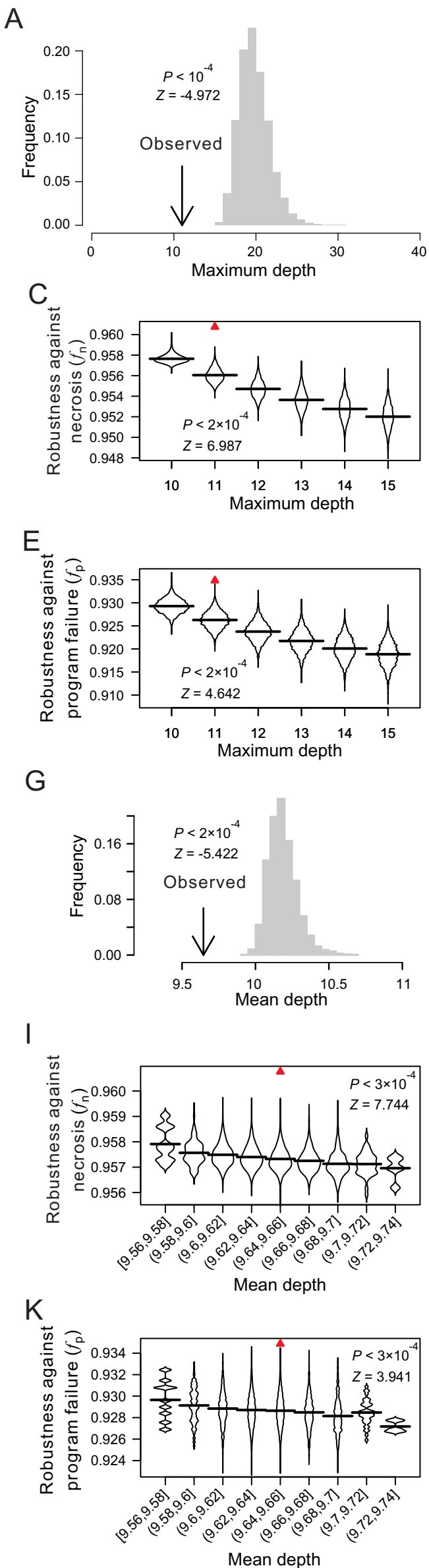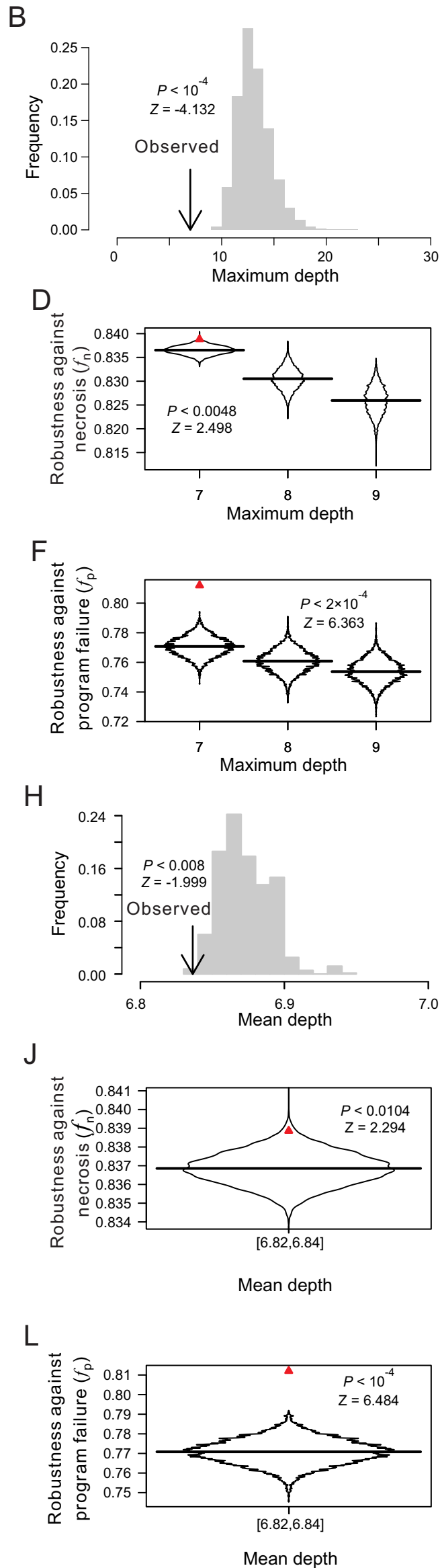

Figure S3

Supplement: Figure S3 — Low depths of terminal cells improve the robustness of the P. marina and H. roretzi lineages to necrosis and program failure. (A–L) These panels are the same as in Fig. 2, except for the species examined. In panels (C)–(F) and (I)–(L), the real lineage is indicated by a red triangle for easy recognition. (PDF) [file pgen.1004501.s003.pdf]

*Pellioditis marina*

*Halocynthia roretzi*

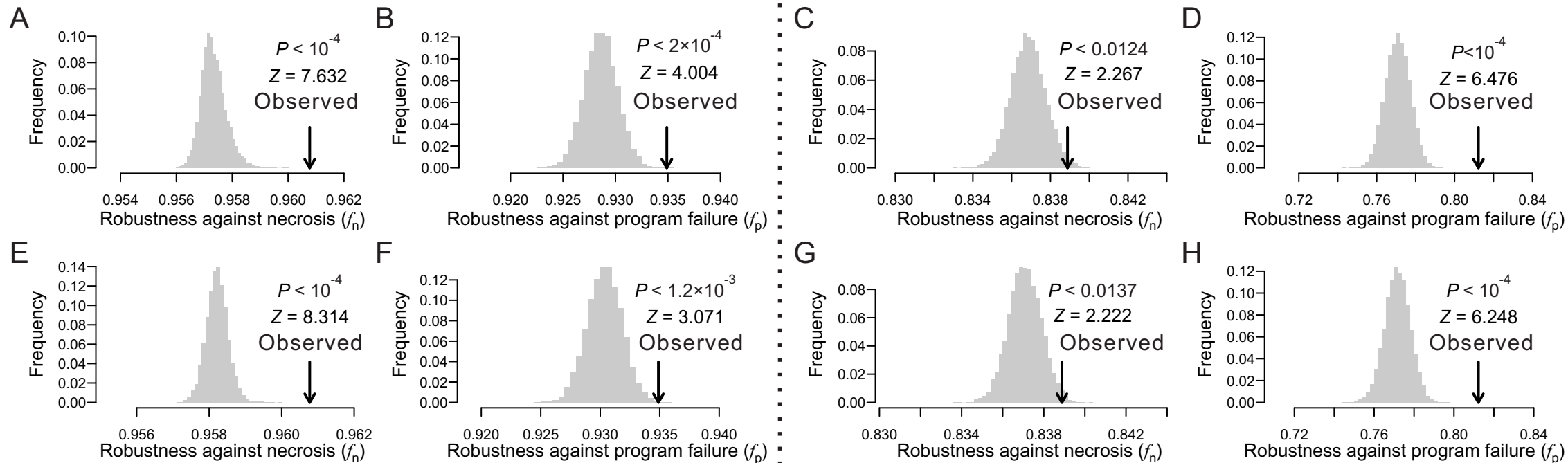

Figure S4

Supplement: Figure S4 — Lineal topology and terminal cell organization contribute to the robustness of the P. marina and H. roretzi lineages. (A–H) These panels are the same as in Fig. 3, except for the species examined. (PDF) [file pgen.1004501.s004.pdf]

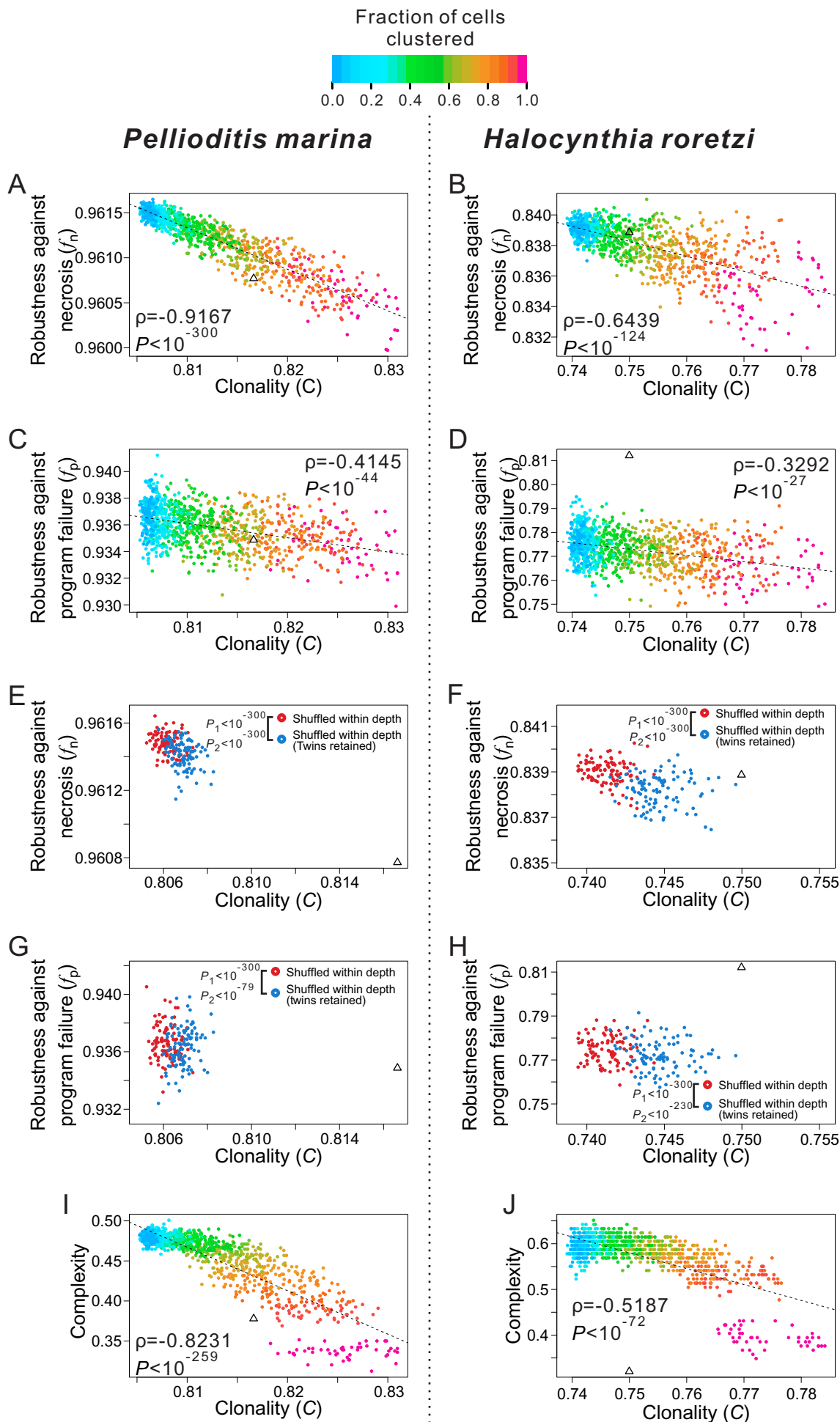

Figure S6

Supplement: Figure S6 — Non-clonality of cell types contributes to the robustness of P. marina and H. roretzi cell lineages. (A–J) These panels are the same as panels A, B, D, E, and F in Fig. 5, except for the species examined. There is no data of between-cell physical distances in P. marina and H. roretzi. Consequently, the analysis in Fig. 5C cannot be conducted for these two species. (PDF) [file pgen.1004501.s006.pdf]

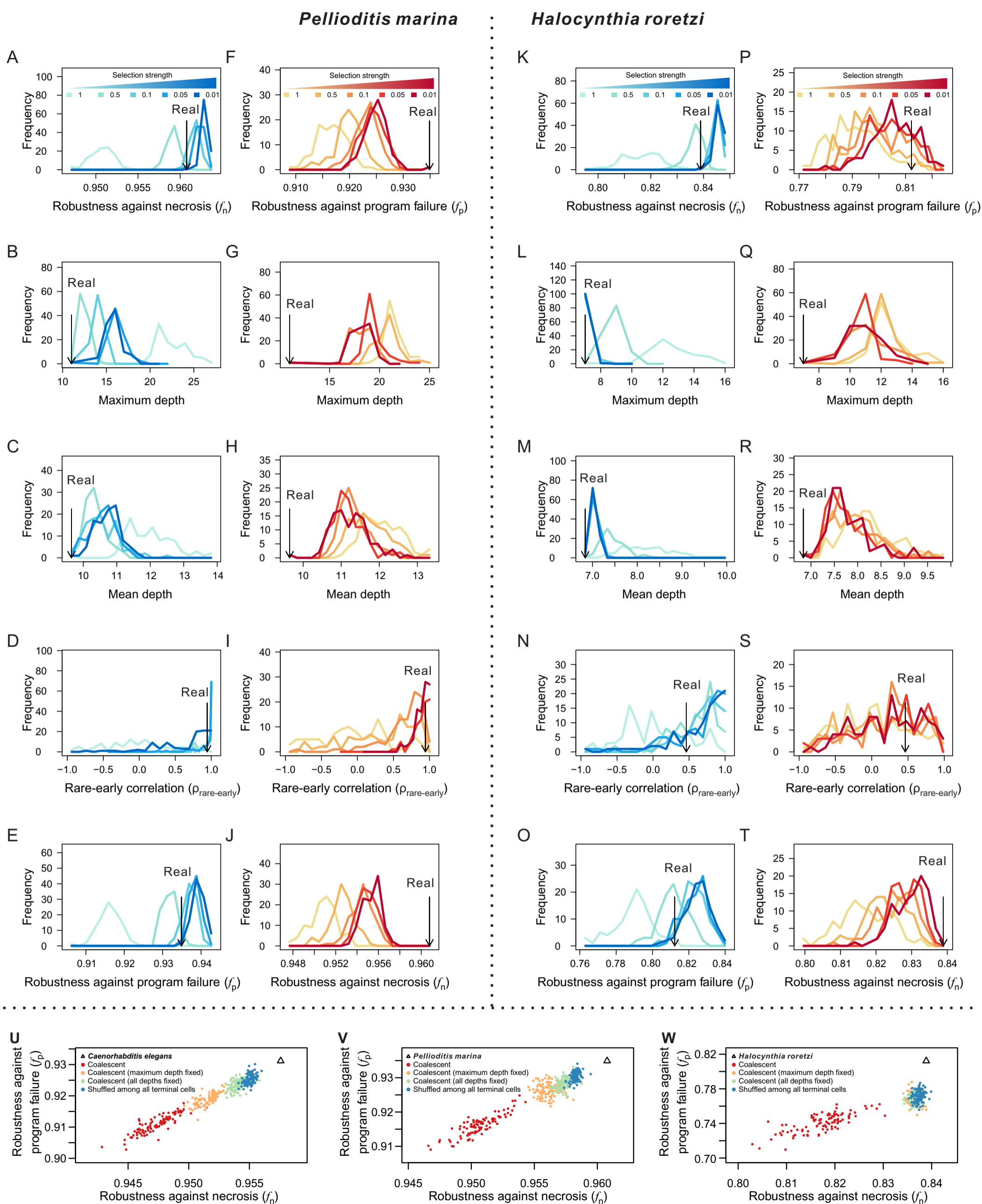

Figure S7

Supplement: Figure S7 — The macroevolution simulations for P. marina and H. roretzi lineages and the correlation between robustness to necrosis (f n) and that to program failure (f p). (A–T) These panels are the same as in Fig. 6, except that the species examined are P. marina and H. roretzi. (U–W) Correlation between f n and f p among various random lineages generated from the real lineages of (U) C. elegans, (V) P. marina, or (W) H. roretzi. The real lineages are indicated by triangles. (PDF) [file pgen.1004501.s007.pdf]

*Pellioditis marina**Halocynthia roretzi*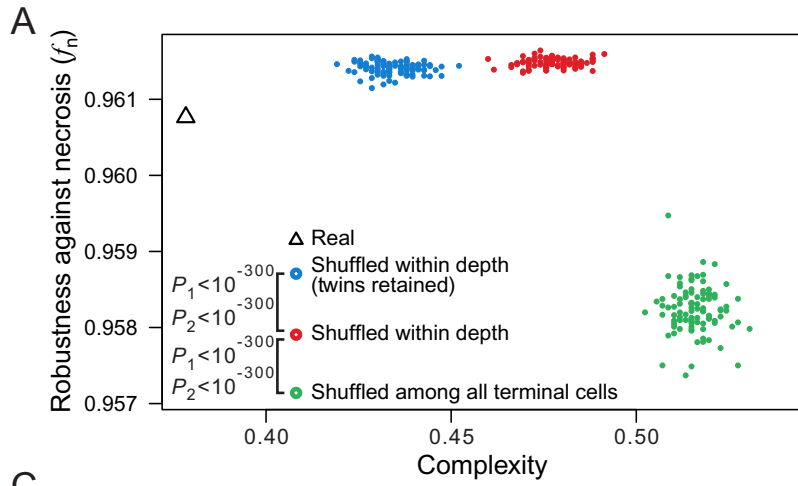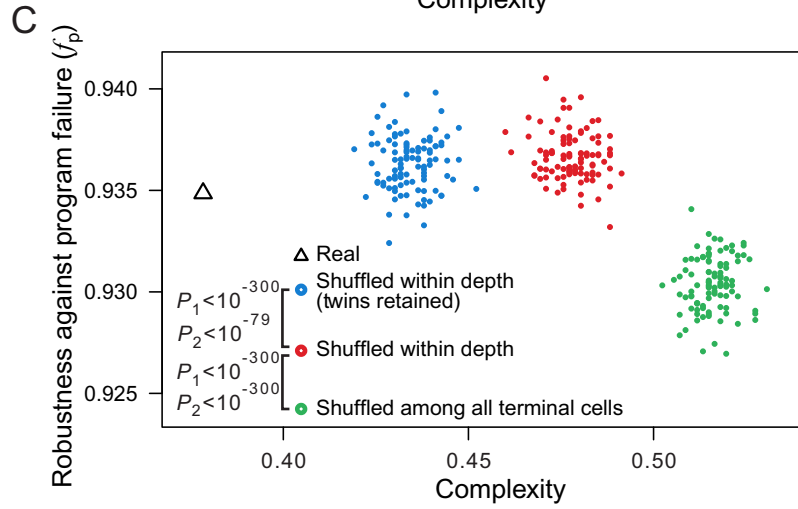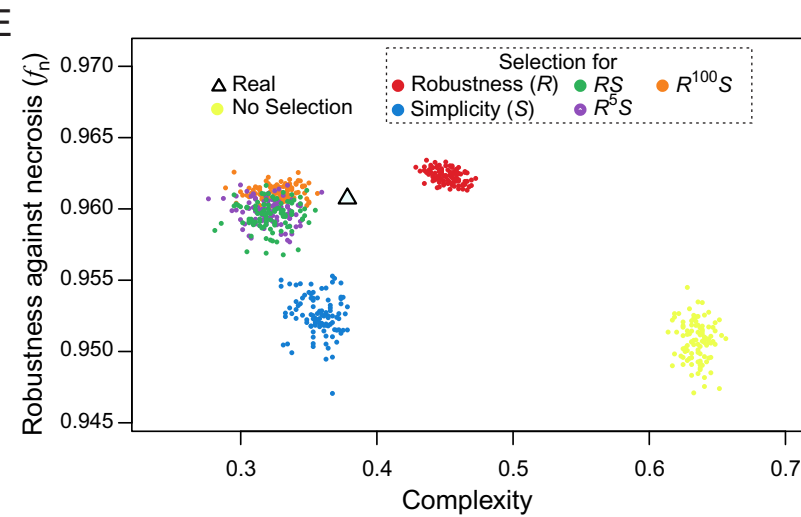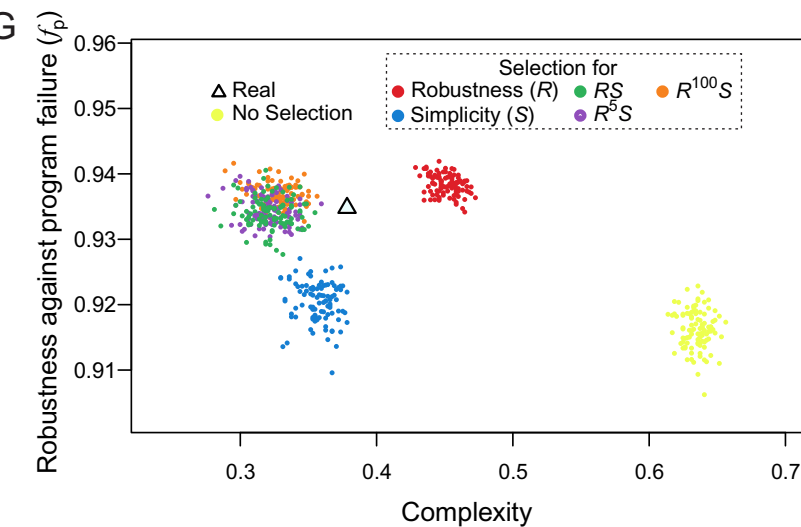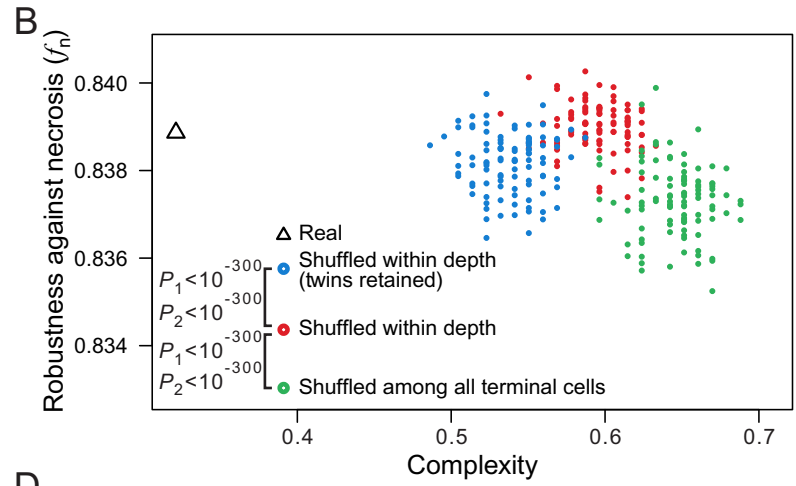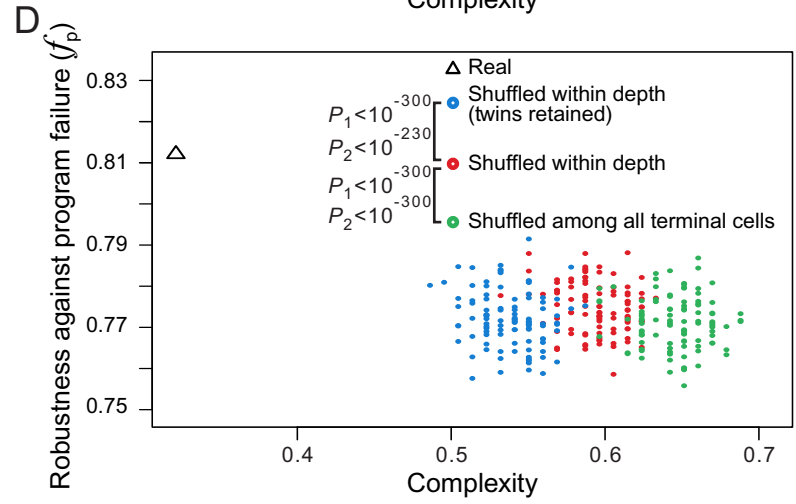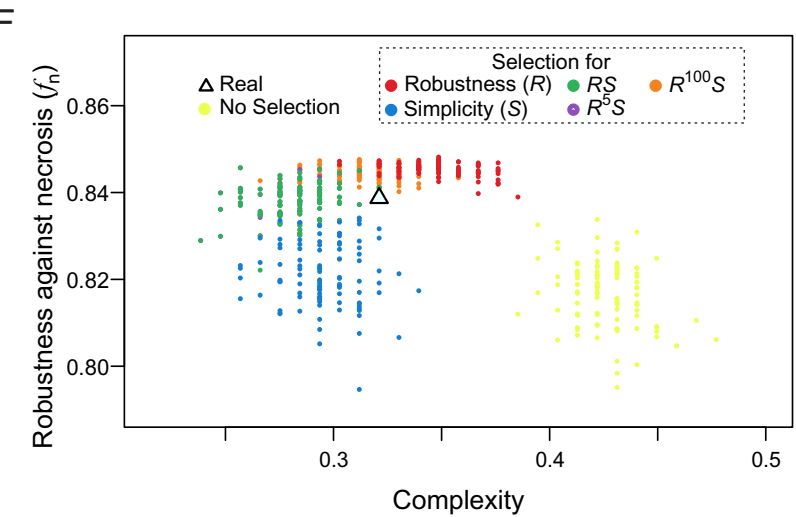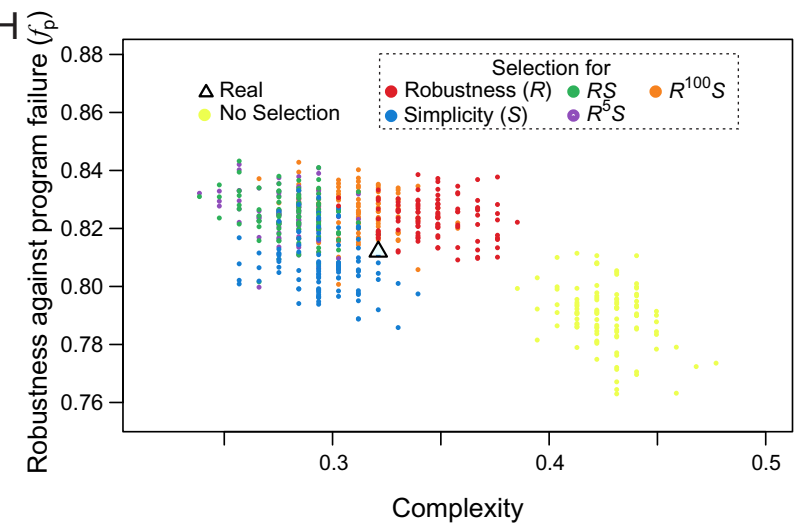

Figure S8

Supplement: Figure S8 — Selection for simplicity cannot explain the robustness of the P. marina and H. roretzi lineages. (A–H) These panels are the same as in Fig. 7, except for the species examined. (PDF) [file pgen.1004501.s008.pdf]
