# Supplementary material for: Determinative Developmental Cell Lineages Are Robust to Cell Deaths
Source: PLoS Genet. 2014 Jul 24;10(7):e1004501. doi: 10.1371/journal.pgen.1004501 (PMC4110091; doi:10.1371/journal.pgen.1004501)
Supplement: Figure S2 — Procedure of generating random lineages by the coalescent process. (PDF) [file pgen.1004501.s002.pdf]

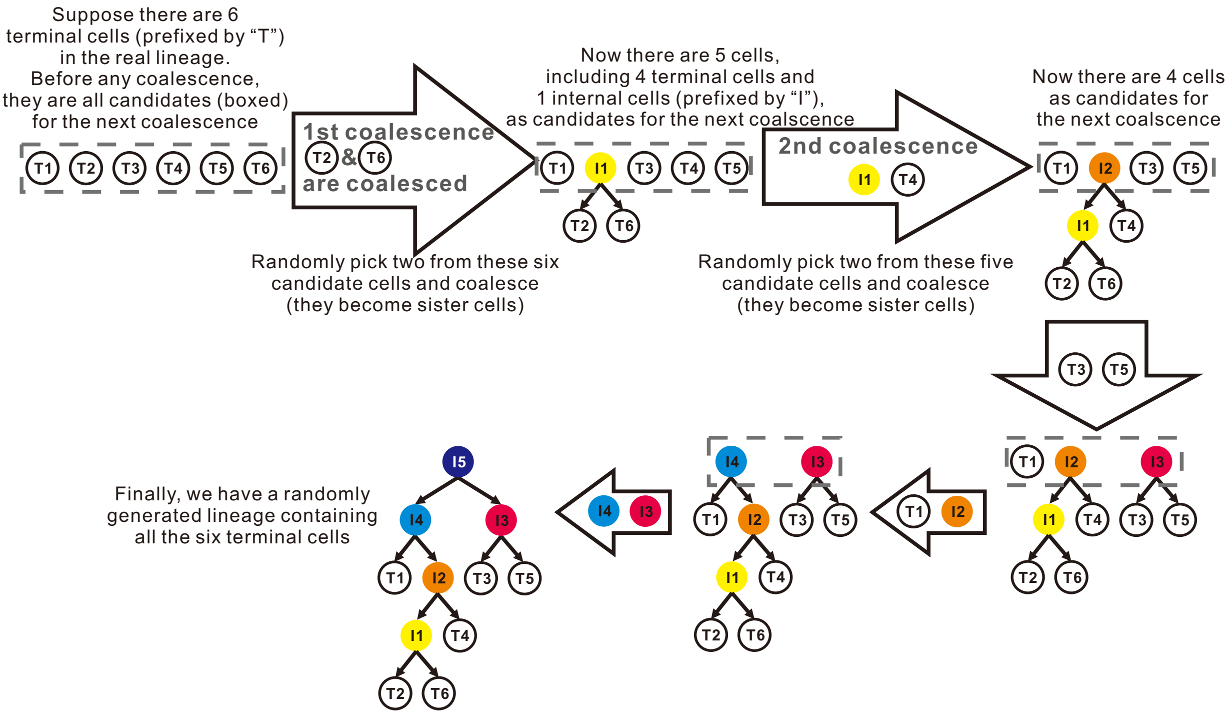
Suppose there are 6 terminal cells (prefixed by "T") in the real lineage. Before any coalescence, they are all candidates (boxed) for the next coalescence
T1 T2 T3 T4 T5 T6
1st coalescence
T2 & T6
are coalesced
Randomly pick two from these six candidate cells and coalesce (they become sister cells)
Now there are 5 cells, including 4 terminal cells and 1 internal cells (prefixed by "I"), as candidates for the next coalscence
T1 I1 T3 T4 T5
T2 T6
2nd coalescence
I1 T4
Randomly pick two from these five candidate cells and coalesce (they become sister cells)
Now there are 4 cells as candidates for the next coalscence
T1 I2 T3 T5
I1 T4
T2 T6
T3 T5
T1 I2 I3
I1 T4 T3 T5
T2 T6
T1 I2
I4 I3
T1 I2 T3 T5
I1 T4
T2 T6
I4 I3
Finally, we have a randomly generated lineage containing all the six terminal cells
I5
I4 I3
T1 I2 T3 T5
I1 T4
T2 T6

Figure S2
