# Supplementary material for: Determinative Developmental Cell Lineages Are Robust to Cell Deaths
Source: PLoS Genet. 2014 Jul 24;10(7):e1004501. doi: 10.1371/journal.pgen.1004501 (PMC4110091; doi:10.1371/journal.pgen.1004501)
Supplement: Figure S5 — That rare cell types tend to have low depths improves the robustness of cell lineages. (A–H) These panels are the same as in Fig. 4, except that the species examined are P. marina and H. roretzi. Cell types in panel (A): Ger, germ; Hyp, hypodermis; Int, intestine; Mus, muscle; Ner, nervous system; Pha, pharynx. Cell types in panel (B): End, endoderm; Epi, epidermis; Mes, mesenchyme; Mus, muscle; Ner, nervous system; Not, notochord. (I) Reclassification of the eight C. elegans cell types based on expression similarity among cells. The total number of terminal cells belonging to each type is given in the parentheses. For a given functional cell type, the fraction of cells belonging to each expression-based cell type is indicated by the area of the circle in the matrix. The mutual information between the two classifications would be 2.33 if they match perfectly. The actual mutual information is 1.45, indicating a substantial difference between the two classifications. (J) Rare-early correlation in C. elegans under the expression-based cell type classification shown in (I). (K) The rare-early correlation in C. elegans under the expression-based cell type classification is robust to the number of cell types classified. In each case, the probability that a random lineage has a higher rare-early correlation than that observed in C. elegans is smaller than 0.001. The probability is determined as in Fig. 4B. (PDF) [file pgen.1004501.s005.pdf]

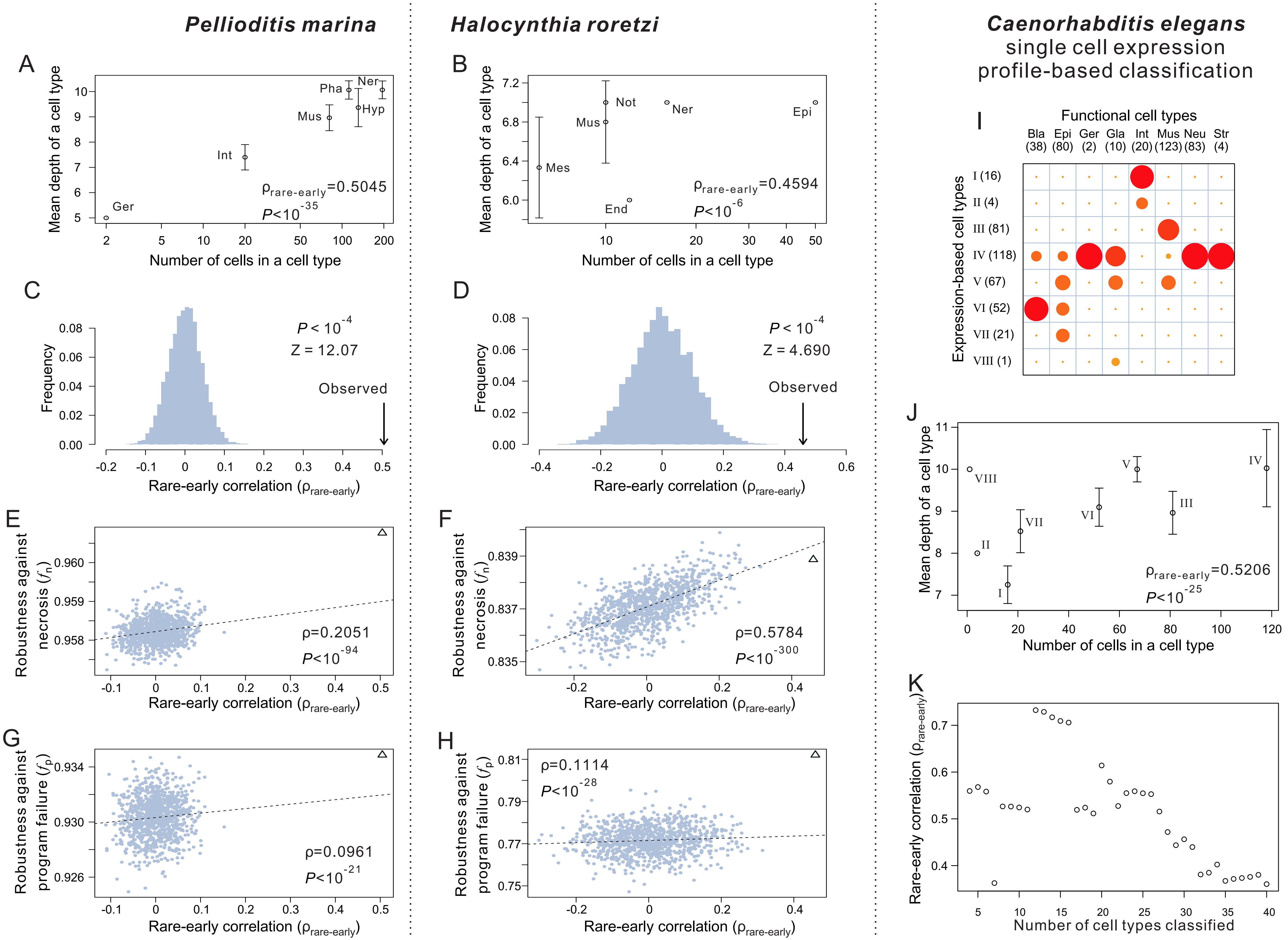
Pellioditis marina
Halocynthia roretzi
Caenorhabditis elegans
single cell expression
profile-based classification
A
Mean depth of a cell type
Number of cells in a cell type
Ger
Int
Mus
Pha
Ner
Hyp
ρrare-early=0.5045
P<10-35
B
Mean depth of a cell type
Number of cells in a cell type
Mes
Mus
Not
Ner
End
Epi
ρrare-early=0.4594
P<10-6
C
Frequency
P < 10-4
Z = 12.07
Observed
Rare-early correlation (ρrare-early)
D
Frequency
P < 10-4
Z = 4.690
Observed
Rare-early correlation (ρrare-early)
E
Robustness against necrosis (fn)
ρ=0.2051
P<10-94
Rare-early correlation (ρrare-early)
F
Robustness against necrosis (fn)
ρ=0.5784
P<10-300
Rare-early correlation (ρrare-early)
G
Robustness against program failure (fp)
ρ=0.0961
P<10-21
Rare-early correlation (ρrare-early)
H
Robustness against program failure (fp)
ρ=0.1114
P<10-28
Rare-early correlation (ρrare-early)
I
Functional cell types
Bla (38) Epi (80) Ger (2) Gla (10) Int (20) Mus (123) Neu (83) Str (4)
Expression-based cell types
I (16)
II (4)
III (81)
IV (118)
V (67)
VI (52)
VII (21)
VIII (1)
J
Mean depth of a cell type
Number of cells in a cell type
ρrare-early=0.5206
P<10-25
K
Rare-early correlation (ρrare-early)
Number of cell types classified

Figure S5
