# Supplementary material for: Determinative Developmental Cell Lineages Are Robust to Cell Deaths
Source: PLoS Genet. 2014 Jul 24;10(7):e1004501. doi: 10.1371/journal.pgen.1004501 (PMC4110091; doi:10.1371/journal.pgen.1004501)
Supplement: Table S1 — Requirements for a developmental cell lineage dataset to be amenable to our analysis. (PDF) [file pgen.1004501.s009.pdf]

Table S1. Requirements for a developmental cell lineage dataset to be amenable to our analysis

Requirements (All must be met)	Descriptions	Consequences if unmet
1	The lineage must have the form of a binary tree.	Mother/daughter relationships are obscure. Note that a fate map is unusable because of the lack of information on intermediate cell divisions.
2	The lineage must start from the zygote and contain all cells up to a developmental stage with at least 100 terminal cells.	A lineages starting from a non-zygote cannot be used to calculate the robustness of the lineage. A lineage with <100 terminal cells does not provide sufficient information for statistical analysis.
3	All the terminal cells at this stage must be included in the lineage data.	Missing terminal cells lead to erroneous estimation of robustness.
4	The terminal cells should be functionally categorized.	The effect of cell deaths cannot be estimated without functional categorization of the terminal cells.
