# Supplementary material for: Determinative Developmental Cell Lineages Are Robust to Cell Deaths
Source: PLoS Genet. 2014 Jul 24;10(7):e1004501. doi: 10.1371/journal.pgen.1004501 (PMC4110091; doi:10.1371/journal.pgen.1004501)
Supplement: Table S2 — Some well-known developmental cell lineage datasets that are not amenable to our analysis. (PDF) [file pgen.1004501.s010.pdf]

Table S2. Some well known developmental cell lineage datasets that are not amenable to our analysis

Species	Database/Reference	**Unmet requirement(s)** Refer to Table S1 for details	Note
Danio rerio (Zebrafish)	http://zfin.org/ and http://www.embl.de/digitalembryo/fish.html<br><br>Keller PJ, et al. 2008. Reconstruction of Zebrafish Early Embryonic Development by Scanned Light Sheet Microscopy. Science 322: 1065–1069.	1, 4	
Drosophila melanogaster (Fly)	http://www.embl.de/digitalembryo/fly.html<br><br>Keller PJ, et al. 2010. Fast, high-contrast imaging of animal development with scanned light sheet-based structured-illumination microscopy. *Nat Meth* **7**: 637–642.	1, 4	
Capitella teleta (Polychaete worm)	Meyer NP, et al. 2010. A comprehensive fate map by intracellular injection of identified blastomeres in the marine polychaete Capitella teleta. EvoDevo 1: 8.	1	
Gallus gallus (Chicken)	Garcia-Lopez R, Pombero A, Martinez S. 2009. Fate map of the chick embryo neural tube. Development, Growth & Differentiation 51: 145–165.	1, 2	Similar for non-zygote based fate maps of several other species
Xenopus laevis (frog)	http://www.xenbase.org/anatomy/atlas.do	1	
Hirudo medicinalis (Leech)	Gline SE, Kuo D-H, Stolfi A, Weisblat DA. 2009. High resolution cell lineage tracing reveals developmental variability in leech. Dev Dyn 238: 3139–3151.	4	
Strongylocentrotus purpuratus (Sea urchin)	http://www.bio.davidson.edu/Courses/genomics/mirror/urchin/u0urnt.htm<br><br>Rubio-Guivernau JL, et al. 2009. Combining sea urchin embryo cell lineages by error-tolerant graph matching. Conf Proc IEEE Eng Med Biol Soc 2009: 5918–5921.	4	
Halicephalobus gingivalis (Nematode)	Houthoofd W, Borgonie G. 2007. The embryonic cell lineage of the nematode Halicephalobus gingivalis (Nematoda: Cephalobina: Panagrolaimoidea). Nematology 9: 573–584.	3	
Rhabditophanes sp. KR 3021 (fam. Alloionematidae) (Nematode)	Houthoofd W, et al. 2008. The embryonic cell lineage of the nematode Rhabditophanes sp. *Int J Dev Biol* **52**: 963–967.	3	
